# Spatial metabolomics reveals skeletal myofiber subtypes

**DOI:** 10.1126/sciadv.add0455

**Published:** 2023-02-03

**Authors:** Lanfang Luo, Wenwu Ma, Kun Liang, Yuefan Wang, Jiali Su, Ruirui Liu, Taoyan Liu, Ng Shyh-Chang

**Affiliations:** ^1^ Institute of Zoology, Chinese Academy of Sciences, Beijing, China.; ^2^ Institute for Stem Cell and Regeneration, Chinese Academy of Sciences, Beijing, China.; ^3^ University of Chinese Academy of Sciences, Beijing, China.; ^4^ Beijing Institute for Stem Cell and Regenerative Medicine, Beijing, China.; ^5^ Department of Life Sciences and Medicine, University of Science and Technology of China, Hefei, China.

## Abstract

Skeletal muscle myofibers are heterogeneous in their metabolism. However, metabolomic profiling of single myofibers has remained difficult. Mass spectrometry imaging (MSI) is a powerful tool for imaging molecular distributions. In this work, we optimized the workflow of matrix-assisted laser desorption/ionization (MALDI)–based MSI from cryosectioning to metabolomics data analysis to perform high–spatial resolution metabolomic profiling of slow- and fast-twitch myofibers. Combining the advantages of MSI and liquid chromatography–MS (LC-MS), we produced spatial metabolomics results that were more reliable. After the combination of high–spatial resolution MSI and LC-MS metabolomic analysis, we also discovered a new subtype of superfast type 2B myofibers that were enriched for fatty acid oxidative metabolism. Our technological workflow could serve as an engine for metabolomics discoveries, and our approach has the potential to provide critical insights into the metabolic heterogeneity and pathways that underlie fundamental biological processes and disease states.

## Teaser

Spatial metabolomics reveals muscle fiber subtypes and an eye muscle-like myofiber in the hindlimb.

## INTRODUCTION

Matrix-assisted laser desorption/ionization (MALDI)–based mass spectrometry imaging (MSI) promises to combine the molecular sensitivity and specificity of MS with the capability to spatially resolve the distribution of molecules within tissues by performing MALDI on every pixel of a tissue section. A few studies have even suggested that MSI instrumentation is almost able to reach single-cell resolution under specific conditions (*1*–*4*). These high spatial resolutions require the optimization of matrix-molecular crystalloids with sublimation and on-tissue chemical derivatization. Although sublimation can achieve nanoscale matrix crystals, it comes at a significant cost on ionization efficiency and molecular sensitivity, thus rendering it incapable of metabolomic profiling but only capable of several targeted metabolites' analysis. On-tissue chemical derivatization in MSI can enhance molecular ion sensitivity and increase lateral spatial resolution, but it is also confined to the measurement of predefined, labeled compounds (*5*–*7*), and thus, it is incapable of metabolomics as well. Nevertheless, MSI workflows at lower spatial resolution have been developed to upgrade the traditional histological classification of tissue samples by adding MSI to analyze the distribution of metabolomic markers that encode for disease states (*8*, *9*). In this aspect, although the field of digital image analysis has rapidly advanced to support the evaluation and annotation of MSI using machine learning algorithms, inaccurate co-registration of images is another obstacle for MSI metabolomics (*10*, *11*). Moreover, because there is no chromatography in MSI, there is no retention time information to assist the separation, identification, and accurate annotation of metabolites for MSI metabolomics. Thus, obtaining accurately annotated, accurately co-registered, high-resolution MS images for metabolomic profiling has remained challenging.

Skeletal muscles afford a unique opportunity to test-drive innovations in MSI, due to its relative simplicity in structure for ease of imaging analysis, the large size of each single myofiber cell to provide sufficient metabolites above the ion limit of detection, and the metabolic heterogeneity among myofibers for richness in information for metabolomic analysis. Each myofiber is actually a single multinucleated cell with an elongated structure, and it is an ideal model for high–spatial resolution analysis by MSI.

Myofibers can be broadly classified as slow- and fast-twitch myofibers. On the basis of differential myosin adenosine triphosphatase (ATPase) expression and oxidative metabolism, there is further classification of fast-twitch fibers into three major subtypes: 2a, 2x, and 2b. These myofiber subtypes have different functional and metabolic properties. Slow-twitch type 1 fibers are more resistant to fatigue for long-term endurance exercise, contract more slowly, and display a higher expression of mitochondrial oxidative enzymes. In contrast, fast-twitch type 2 fibers rely more on glycolytic enzymes to rapidly generate energy for quick bursts of resistance exercise, with type 2b being the most glycolytic and type 2a being the least glycolytic, and each subtype has their own unique isoform of myosin heavy chain (*12*).

Metabolomic analysis of myofibers would improve our molecular understanding of all myofiber subtypes and their association with exercise performance, locomotion, sarcopenia (*13*, *14*), cachexia, muscle wasting, and muscle-associated metabolic diseases (*15*, *16*). Furthermore, under the influence of intrinsic and extrinsic factors, myofiber fates can be transformed from fast twitch to slow twitch or vice versa (*17*–*19*). Understanding the dynamic metabolic state of myofibers is helpful to understand the mechanism of fiber remodeling under physiological and pathological conditions as a model for postmitotic cell fate transitions. However, our understanding has been limited by rudimentary biochemical techniques, e.g., fast/slow myosin ATPase immunostaining, or enzyme histochemistry for reduced form of nicotinamide adenine dinucleotide hydrogen (NADH) tetrazolium reductase, succinate dehydrogenase, and cytochrome c oxidase, which are nonquantitative and low-dimensional techniques where only a few enzymes can be analyzed at a time. A few studies have discussed the possibility of metabolic analysis of slow- and fast-twitch fibers, and some have studied single, isolated myofibers ex vivo (*20*–*23*). However, there are still many technical obstacles to overcome, and none have performed metabolomic analysis of myofibers in vivo hitherto.

With these challenges in mind, we optimized the workflow of MALDI-MSI from cryosectioning to MSI metabolomics to analyze in vivo myofibers. We succeeded in characterizing the metabolomic profiles of different myofiber subtypes and discovered a new myofiber subtype in the process, as a proof of principle that our technological workflow can be an engine for metabolic discoveries in muscle biology.

## RESULTS

### Metabolomic fingerprints detected by MALDI- and atmospheric pressure–MALDI-MSI show high reproducibility

**Fig. 1.**
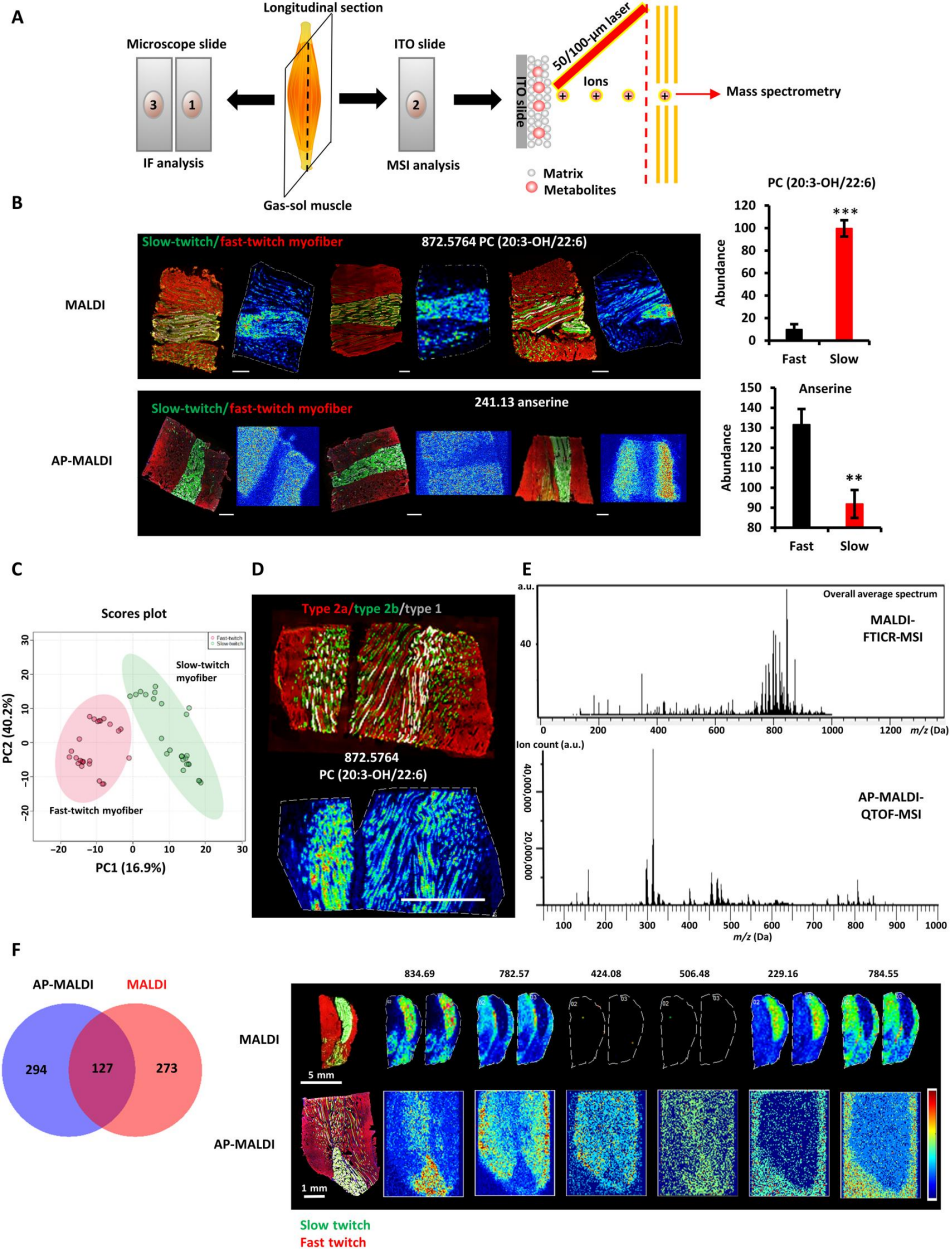
Reproducibility of metabolomic fingerprints of slow- and fast-twitch myofibers in MSI. (**A**) Schematic of the MSI workflow. Every three consecutive sections from the muscle were prepared as a group, with the first and third sections used for immunofluorescence (IF) analysis and the middle sections used for MSI analysis. (**B**) Fingerprint metabolites of slow- and fast-twitch myofibers detected by AP-MALDI-QTOF and MALDI-FTICR (left). Multicolor immunofluorescence staining of the GAS-SOL muscle (right). Means ± SEM; *N* = 3; **P < 0.01 and ***P < 0.001 compared with fast-twitch myofibers; Student’s *t* test. Green, slow twitch; red, fast twitch. Scale bars, 500 μm. (**C**) PLS-DA of the fast-twitch myofibers and the slow-twitch myofibers (*N* = 24 independent MSI experiments). (**D**) Performance of single-cell MSI on myofibers at the high spatial resolution of 25 μm. Immunofluorescence staining (top) and MSI analysis (bottom) of adjacent longitudinal sections of the hindlimb’s GAS-SOL muscle. Green, type 2a myofiber; red, type 2b myofiber; white, type 1 myofiber. Scale bar, 1000 μm. (**E**) Averaged mass spectrum from MALDI (top) and AP-MALDI (bottom) of the GAS-SOL muscle.ITO, indium tin oxide; a.u., arbitrary units. (**F**) Root mean square–normalized abundance and distributions of discriminative peaks (table S1) in slow- and fast-twitch myofibers according to MALDI- and AP-MALDI-MSI of GAS-SOL muscle (left). Fingerprint metabolites of slow- and fast-twitch myofibers detected by AP-MALDI-QTOF and MALDI-FTICR. Immunofluorescence staining and MSI of adjacent longitudinal sections of the hindlimb’s GAS-SOL muscle (right). Green, slow twitch; red, fast twitch.

Skeletal muscle myofibers are classified as slow-twitch (type 1) and fast-twitch fibers (type 2). Slow-twitch fibers are typically dense in mitochondria to allow high oxidative capacity and sustain the energy demands of endurance exercise, whereas fast-twitch fibers typically have less mitochondria and higher levels of glycolytic enzymes to allow rapid bioenergetics and bursts of resistance exercise. Thus, slow oxidative and fast glycolytic myofibers are markedly different in their metabolic states. To test whether MSI can reproducibly and stably classify distinct metabolic states, we studied slow- and fast-twitch myofibers as a model system. We performed MSI of hindlimb gastrocnemius-soleus (GAS-SOL) muscle cryosections by using MALDI and atmospheric pressure (AP)–MALDI (Fig. 1A and Materials and Methods). Multicolor immunofluorescence staining showed that the mouse SOL consists of a majority of type 1 and a minority of type 2a muscle fibers, while the mouse gastrocnemius muscle consists of a majority of fast-twitch myofibers (types 2a, 2x, and 2b) and a minority of slow-twitch myofibers (type 1) (Fig. 1B). Anserine, a known fingerprint metabolite of fast-twitch myofibers (*24*), was found to be highly abundant in the fast-twitch myofibers. In contrast, phosphatidylcholine(PC) (20:3-OH/22:6) and several acylcarnitines were highly abundant in the slow-twitch myofibers (Fig. 1B and fig. S1). To assess the reproducibility of MSI, 24 independent MSI experiments were examined. After processing all of the MSI data with SCiLS Lab (*5*), which accounted for baseline removal, normalization, and peak picking, a total of 1486 mass/charge ratio (*m*/*z*) ion peaks were found. Partial least squares–discriminant analysis (PLS-DA) with the *m*/*z* peaks revealed a tight clustering within fast-twitch (*N* = 24) and slow-twitch myofiber samples (*N* = 24) (Fig. 1C). Among all the *m*/*z* peaks, intraclass correlation coefficient (ICC) analysis revealed that the median ICC values were 0.858 and 0.885 for fast-twitch (*N* = 24) and slow-twitch myofibers (*N* = 24), respectively. Furthermore, we used area under the receiver operating characteristic (ROC) curve (AUC) analysis to define discriminating peaks that we could use to calculate ICCs. A total of 196 discriminative peaks were found (AUC ≥ 0.7) (table S1). A total of 92.8% of these discriminative peaks had ICC values of >0.6 (table S1). Thus, we concluded that the metabolomic fingerprints detected by MSI were reproducible.

To further confirm the fingerprint metabolites of fast- and slow-twitch myofibers, we selected three *m*/*z* features (758.60, 806.80, and 834.65) with interesting spatial distributions and high abundance in myofibers for on-tissue tandem mass spectrometry (MS/MS or MS2) analysis. *m/z* values of 758.60 and 806.80 are biomarkers of fast-twitch myofibers, whereas 834.65 is a biomarker of slow-twitch myofibers (fig. S2 and tables S1 and S2). After on-tissue MS2 and analysis of sufficient fragment spectra with PeakView software (*25*, *26*), these ions were annotated by matching their spectra to metabolite spectra in the Human Metabolome Database (HMDB) and METLIN. 758.60, 806.80, and 834.65 were identified to be phosphatidylethanolamine (PE)(P-16:0/22:0), triglyceride (TG)(14:0/15:0/18:2), and phosphatidic acid (PA)(20:0/24:0), respectively. PA is a metabolic precursor for many lipids and is involved in regulating muscle insulin sensitivity by directly stimulating mammalian target of rapamycin (mTOR), mitogen-activated protein kinase, protein kinase C, and protein kinase D signaling (*27*).

Myofibers are larger than usual cells. It was previously reported (*28*) that the Feret diameter of myofibers ranges from 26.47 to 58.59 μm, and the mean fiber cross-sectional area of myofibers ranges from 539.86 to 1319.50 μm^2^. The minimum and maximum Feret diameters of the smaller oxidative type 1 and type 2a myofibers in mouse SOL are 28.47 ± 0.77 to 47.51 ± 1.73 μm and 26.46 ± 0.69 to 41.76 ± 1.36 μm, respectively. These measurements suggest that a spatial resolution of 25 μm could analyze myofibers at near–single-cell resolution. To further test whether the longitudinal cryosections of myofibers can be analyzed at single-cell level, MSI at the high spatial resolution of 25 μm was performed. We were surprised to find that single type 2a or type 1 myofibers could be identified among the type 2b myofibers using high-resolution MSI profiles (Fig. 1D). This indicated that the metabolomic profiles of myofiber type at single myofiber resolution could be achieved.

AP-MALDI is a softer ionization technique compared with conventional vacuum MALDI, thus increasing the ease of sample preparation and allowing for the analysis of volatile small molecules. To investigate the differences between MALDI and AP-MALDI in slow- and fast-twitch myofibers, we compared the average mass spectrum of the hindlimb muscles detected by MALDI and AP-MALDI. The peaks of MALDI were clustered in the 700- to 900-Da interval. Among the 400 metabolites that were significantly different between slow- and fast-twitch fibers in positive ion mode [2,5-dihydroxybenzoic acid (DHB) matrix], 83.8% were lipids. In contrast, the peaks of AP-MALDI were spread across the 100- to 1000-Da interval in positive ion mode [1,5-diaminonaphathelene (1,5-DAN) matrix]. Among the 421 metabolites that were significantly different between slow- and fast-twitch fibers, 91.2% were lipids (Fig. 1E and table S2). A Venn diagram analysis of the differential metabolites found in AP-MALDI and MALDI data of the myofibers showed that 127 metabolites overlapped or ~31% of the differential metabolites detected in both AP-MALDI and MALDI. These 127 metabolites included lipids, anserine, carnosine, and many acylcarnitines (Fig. 1F and table S2). Thus, both MALDI-MSI and AP-MALDI-MSI are capable of distinguishing important differences (common trends) between slow- and fast-twitch myofibers, but each with its own unique range of detection, and a combination of both MSI techniques can cover a wider range of metabolites.

### A workflow that combines liquid chromatography–MS and MSI for metabolomic analysis

**Fig. 2.**
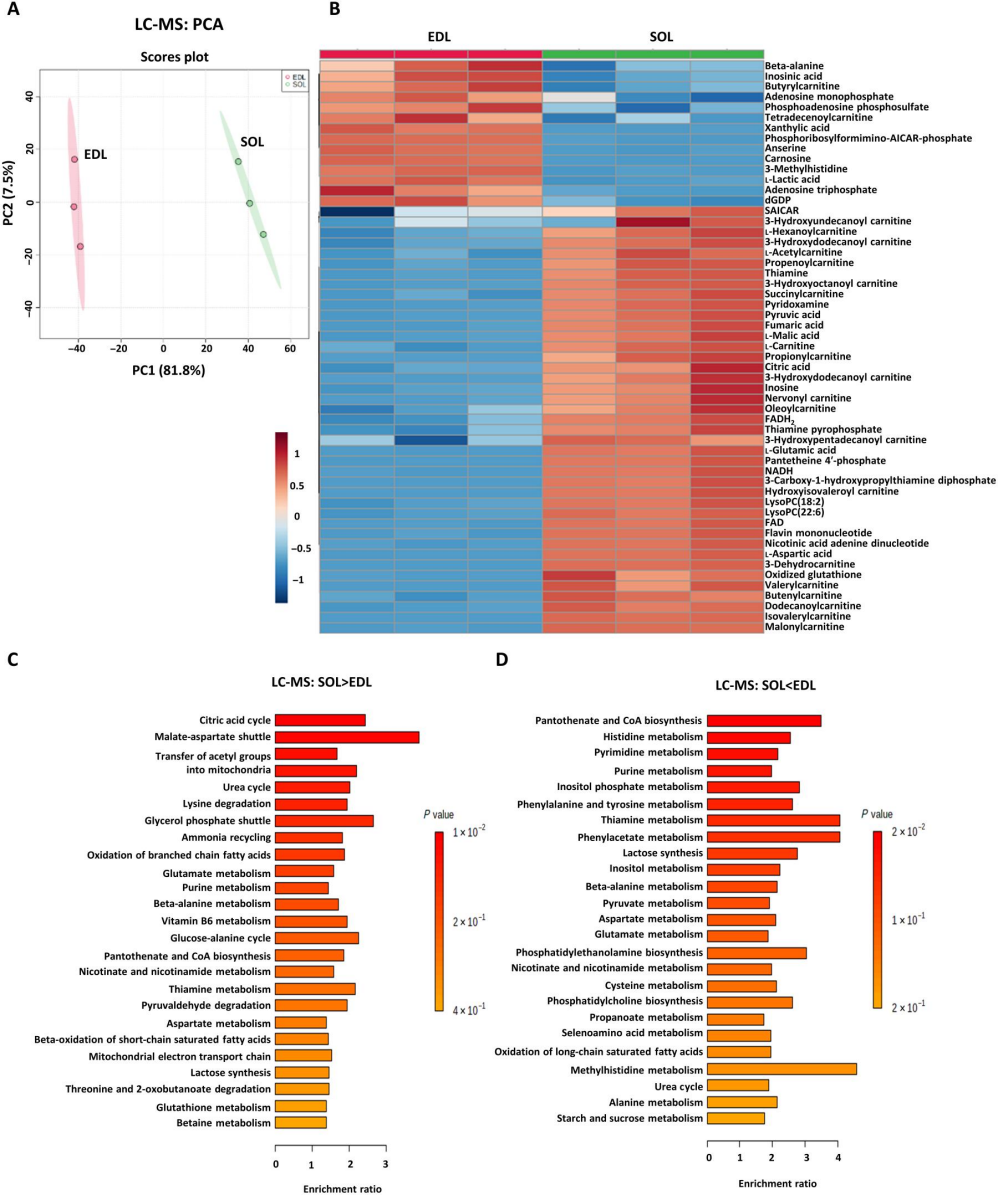
LC-MS metabolomic analysis of slow- and fast-twitch myofibers. (**A**) PCA of the fast-twitch EDL and the slow-twitch soleus (SOL) muscles from different animals (*N* = 3). The two PCs explaining the largest parts of the data variation are shown. (**B**) Heatmap of differential metabolites (*P* < 0.05, *N* = 3, Student’s *t* test). Heatmap denotes the metabolite signal intensities of each sample, showing significantly altered metabolites that increased and decreased in the EDL, relative to the SOL muscle. (**C** and **D**) Metabolite set enrichment analysis (MSEA) of differentially abundant metabolites in EDL and SOL muscles based on LC-MS data. (C) MSEA of significantly up-regulated metabolites in SOL muscles. Citric acid cycle: *P* = 0.012, *N* = 3; malate-aspartate shuttle: *P* = 0.014, *N* = 3. (D) MSEA of significantly up-regulated metabolites in EDL muscles. Pantothenate and coenzyme A (CoA) biosynthesis: *P* = 0.024, *N* = 3; histidine metabolism: *P* = 0.026, *N* = 3; pyrimidine metabolism: *P* = 0.037, *N* = 3; purine metabolism: *P* = 0.042, *N* = 3; inositol phosphate metabolism: *P* = 0.049, *N* = 3. Bonferroni-corrected *P* values were calculated using the Quantitative Enrichment Analysis (QEA) module of MetaboAnalyst MSEA based on the globaltest algorithm and a generalized linear model to estimate the *Q*-stat for each metabolite set.

To explore the metabolic states of slow- and fast-twitch myofibers accurately at the metabolomic level, the metabolomic profiles of fast-twitch EDL and slow-twitch SOL muscles were characterized by both liquid chromatography–MS (LC-MS) and MSI (see Materials and Methods). After processing the LC-MS data with Waters Progenesis QI (*29*, *30*), which accounted for adducts, fragments, and isotope peaks and searched against the HMDB library, a total of 24,945 RT-*m*/*z* ion peaks, 9222 peaks in negative ion mode, and 15,723 peaks in positive ion mode were found, and a total of 11,523 ion peaks were identified. Principal components analysis (PCA) of the ion peaks revealed a tight clustering within EDL and SOL samples (Fig. 2A) and a clear separation between them in both positive ion mode and negative ion mode (fig. S3, A and B). Quality control sample results further proved that the LC-MS metabolomics method had excellent reproducibility (fig. S3, A and B).

To definitively identify the LC-MS fingerprint metabolites of slow- and fast-twitch myofibers, PLS-DA was applied followed by filtering for metabolites with loading scores of ≥0.05 and Variable Importance in the Project (VIP) scores of ≥1 (fig. S3, C to E). Using these cutoff values, 34 metabolites were identified as potential biomarkers of EDL and SOL muscles, of which the most significant metabolites included anserine and carnosine for EDL and pyruvic acid and flavin adenine dinucleotide (FAD) for SOL (table S3). Using MetaboAnalyst to perform detailed statistical analysis and hierarchical clustering of the LC-MS data, 738 differential metabolites were found to be significantly altered between EDL and SOL muscles, where anserine, carnosine, lactic acid, inosinic acid, xanthylic acid, deoxyguanosine diphosphate, adenosine monophosphate (AMP), adenosine triphosphate, phosphoadenosine phosphosulfate, and phosphoribosyl-formimino-aminoimidazole-carboxamide ribonucleotide (AICAR)-phosphate were highly abundant in the glycolytic EDL. Anserine and carnosine are both dipeptides containing methylhistidine, which are known to be higher in animals with sprinter characteristics and thought to manifest antioxidant properties (Fig. 2B) (*31*). In contrast, mitochondrial tricarboxylic acid (TCA) cycle–related metabolites, including pyruvic acid; malic acid; fumaric acid; aspartic acid; glutamic acid; glutamine; ADP; and derivatives of oxidative phosphorylation (OxPhos)–related cofactors such as pyridoxamine (vitamin B6), pantetheine 4′-phosphate (vitamin B5), NADH (vitamin B3), and FAD (vitamin B2) were highly abundant in the oxidative SOL (Fig. 2B). In addition, mitochondrial fatty acid oxidation (FAO)–related metabolites including carnitine, acetylcarnitine, and a series of acylcarnitines were highly abundant in SOL muscles.

Metabolite set enrichment analysis (MSEA) of the 738 metabolites showed that 10 pathways were significantly enriched in SOL muscles, including the TCA cycle, malate-aspartate shuttle, and glycerol phosphate shuttle for transferring reducing equivalents into mitochondria (Fig. 2C), and four pathways were significantly enriched in EDL muscles, including histidine metabolism, pyrimidine metabolism, and purine metabolism (Fig. 2D).

**Fig. 3.**
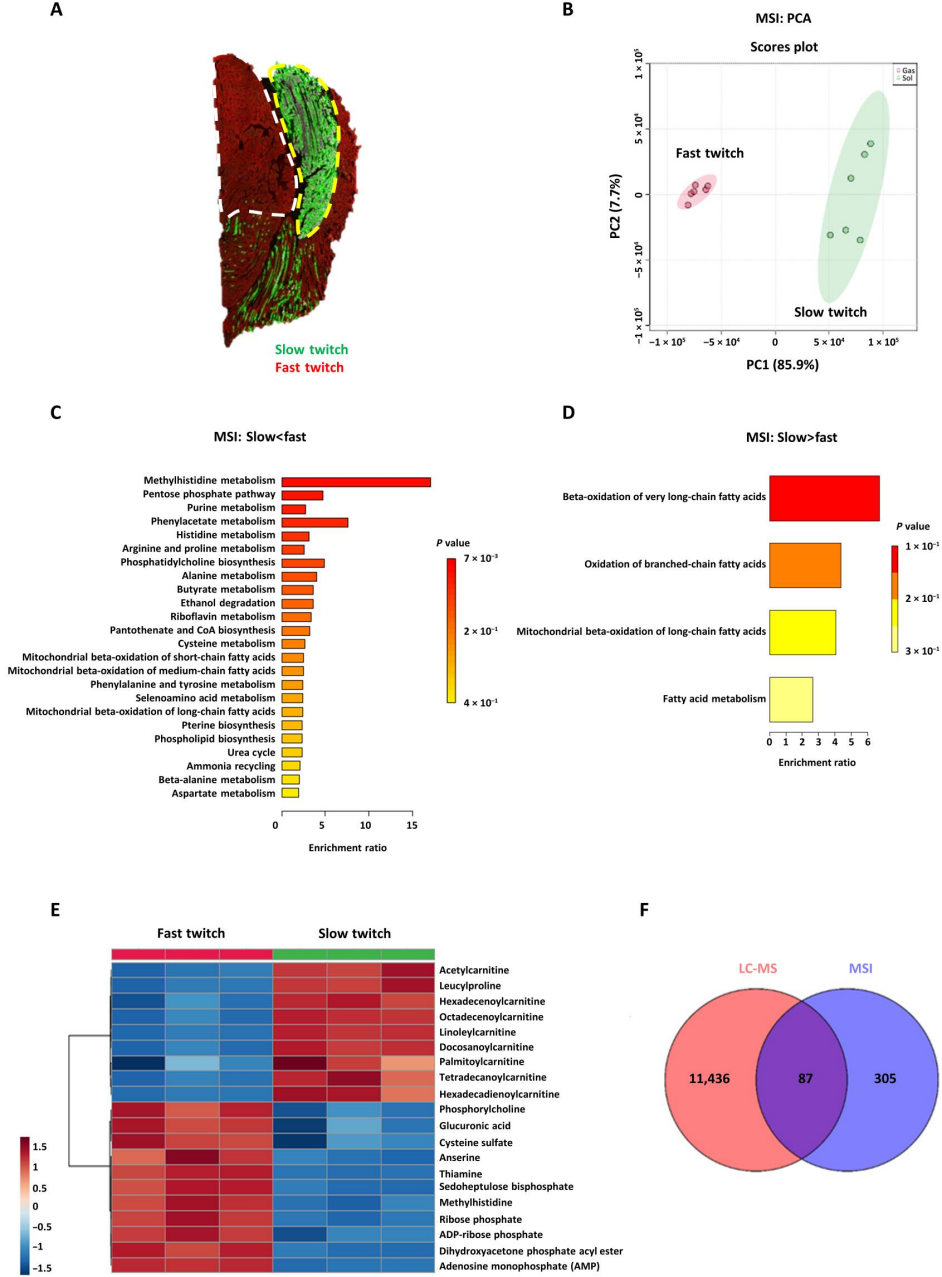
Automated MSI analysis of metabolic patterns in slow- and fast-twitch myofibers. (**A**) Representative immunofluorescence images. Immunofluorescence staining of slow- and fast-twitch myofibers in the GAS-SOLmuscles. Red, fast-twitch myofibers; green, slow-twitch myofibers; white dotted line, ROI with fast-twitch myofibers; yellow dotted line, ROI with slow-twitch myofibers. (**B**) PCA of slow-twitch (green) and fast-twitch (red) myofiber regions (*N* = 6 each) based on MSI data. (**C** and **D**) MSEA of differentially abundant metabolites in slow- and fast-twitch myofibers based on MSI data. (C) MSEA of up-regulated metabolites in fast-twitch myofibers. Methylhistidine metabolism: *P* = 0.057, *N* = 6; PPP: *P* = 0.065, *N* = 6; purine metabolism: *P* = 0.088, *N* = 6. (D) MSEA of up-regulated metabolites in slow-twitch myofibers, beta-oxidation of very long-chain fatty acids: *P* = 0.14, *N* = 6. Bonferroni-corrected *P* values (cutoff *P* < 0.15) were calculated using the QEA module of MetaboAnalyst MSEA, based on the globaltest algorithm and a generalized linear model to estimate the *Q*-stat for each metabolite set. (**E**) Heatmap of differential metabolites (*P* < 0.05, *N* = 3, Student’s *t* test). The heatmap denotes the metabolite signal intensities of each sample, showing significantly altered metabolites that increased and decreased in the fast-twitch myofibers, compared with the slow-twitch myofibers. (**F**) Venn diagram analysis to compare the degree of overlap between LC-MS and MSI data.

To verify whether MSI could automatically identify these metabolic patterns in complex samples in a spatial metabolomics pipeline, GAS-SOL muscle cryosections were analyzed with MSI. GAS-SOL muscles can be further divided into the slow- and fast-twitch myofiber regions of interest (ROIs) according to immunofluorescence staining (Fig. 3A). Region-specific mass spectra were extracted on the basis of the overlay and co-registration of immunofluorescence and computationally reconstructed MS images. A PCA plot presented a clear separation between the slow- and fast-twitch myofiber regions (Fig. 3B). Using statistical analysis packages in MetaboAnalyst and online metabolomics databases (HMDB and METLIN), 392 metabolites were found to show significant differences between the two myofiber groups. MSEA showed that, similar to the LC-MS data, methylhistidine metabolism and both anserine and methylhistidine, in particular, were enriched in the MSI data of fast-twitch myofibers (Fig. 3, C and E). Glycolysis-, pentose phosphate pathway (PPP)–, and purine-related metabolites such as dihydroxyacetone phosphate acyl ester (intermediate between glycolysis and phospholipid synthesis), sedoheptulose bisphosphate (intermediate between glycolysis and PPP), ribose phosphate (product of PPP), AMP, and adenosine diphosphate (ADP)–ribose phosphate were also up-regulated in fast-twitch myofibers (Fig. 3, C and E). In contrast, beta-oxidation of fatty acids was enriched in slow-twitch myofibers, and both acetylcarnitine and many acylcarnitines were significantly up-regulated in slow-twitch myofibers (Fig. 3, D and E). A Venn diagram analysis of the differential metabolites based on LC-MS and MSI data of the myofibers showed that 87 metabolites overlapped, including carnitine, acetylcarnitine, many acylcarnitines, carnosine, anserine, methylhistidine, histidine, and AMP (Fig. 3F and table S3).

### Increased lipid uptake and oxidation are metabolic hallmarks of oxidative myofibers

**Fig. 4.**
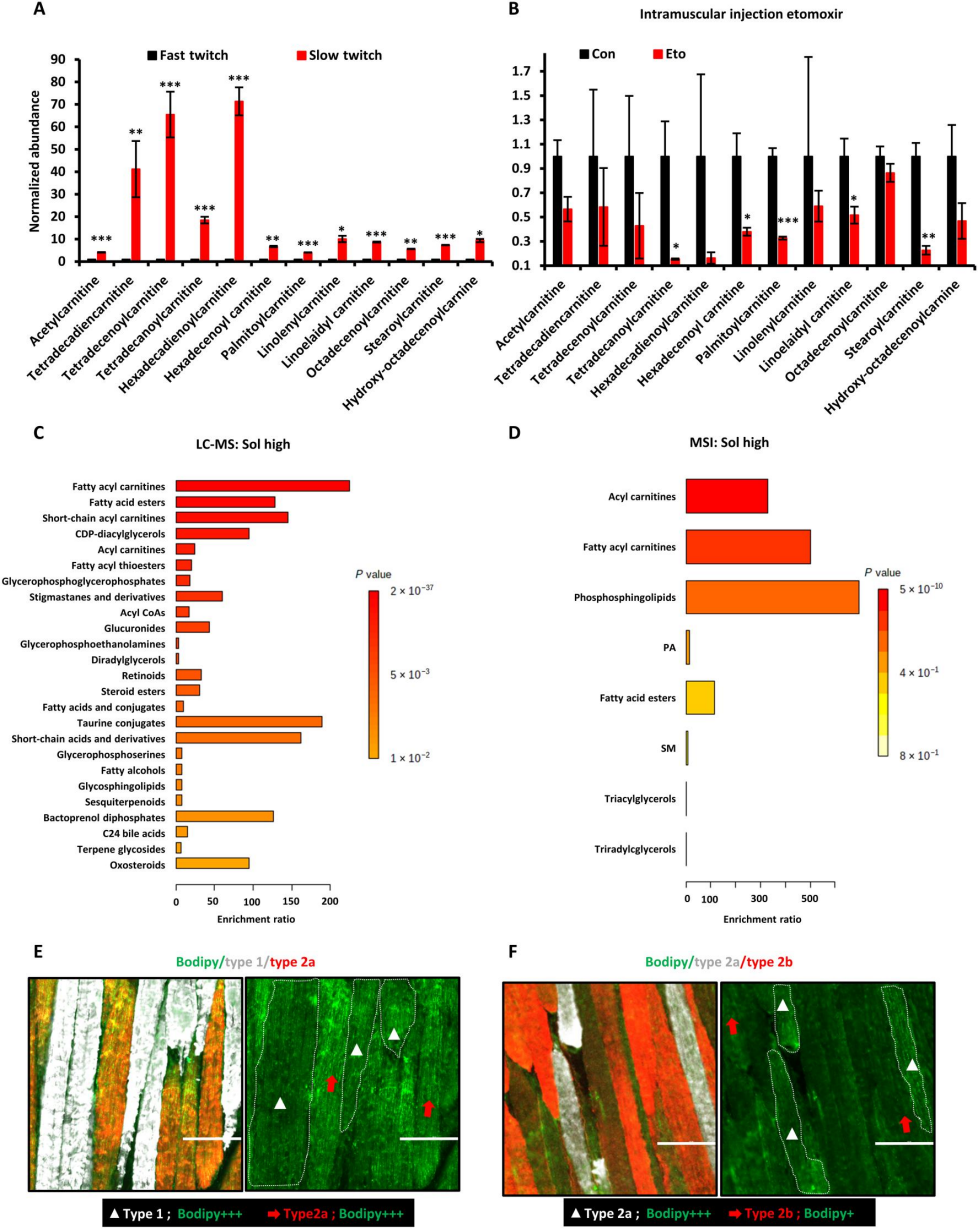
Increased lipid uptake and FAO are hallmarks of oxidative myofibers. (**A**) Relative abundance of acylcarnitines in slow-twitch myofibers, relative to fast-twitch myofibers, based on MSI data (means ± SEM; *P < 0.05, **P < 0.01, and ***P < 0.001 compared with fast-twitch myofiber; Student’s *t* test). (**B**) Relative abundance of acylcarnitines at 30 min after intramuscular injection of the CPT1 inhibitor etomoxir into the gastrocnemius muscle, based on MSI data (means ± SEM; *P < 0.05, **P < 0.01, and ***P < 0.001 compared with fast-twitch myofiber; Student’s *t* test). (**C** and **D**) MSEA^Lipid^ of differentially abundant lipids based on (C) LC-MS data of slow-twitch myofibers, relative to fast-twitch myofibers (fatty acyl carnitines: *P* < 0.001; fatty acid esters: *P* < 0.001; short-chain acyl carnitines: *P* < 0.001; cytidine diphosphate (CDP)-diacylglycerols: *P* < 0.001; acyl carnitines: *P* < 0.001; fatty acyl thioesters: *P* < 0.001), and (D) MSI data of SOL muscles, relative to EDL muscles (acyl carnitines: *P* < 0.001; fatty acyl carnitines: *P* < 0.001; phosphosphingolipids: *P* = 0.002; fatty acid esters: *P* = 0.012.). Bonferroni-corrected *P* values were calculated using the QEA module of MetaboAnalyst MSEA, based on the globaltest algorithm and a generalized linear model to estimate the *Q*-stat for each metabolite set. (**E**) Immunofluorescence staining for different oxidative subtypes of myofibers and intramyocellular lipid droplets with the Bodipy dye. White, type 1; red, type 2a; green, Bodipy dye. Scale bars, 100 μm. (**F**) Immunofluorescence staining for type 2a and type 2b myofibers and intramyocellular lipid droplets with the Bodipy dye. White, type 2a; red, type 2b; green, Bodipy dye. Scale bars, 100 μm.

The LC-MS and MSI data indicated that slow-twitch oxidative myofibers have a high abundance of acylcarnitines. To validate whether FAO intermediates are accumulating in slow-twitch oxidative myofibers because of reduced FAO flux or increased FAO flux, we compared acylcarnitine levels in fast-twitch versus slow-twitch myofibers before and after FAO inhibition. MS images of the hindlimb GAS-SOL muscle cryosections indicated that the abundance of acetylcarnitine and acylcarnitines was significantly higher in slow-twitch myofibers than in fast-twitch myofibers (Fig. 4A and fig. S1A). After intramuscular injection of etomoxir, a specific inhibitor of FAO through its irreversible inhibition of carnitine palmitoyl-transferase 1 (CPT1), the high levels of acetylcarnitine and many acylcarnitines in slow-twitch myofibers were decreased compared to the vehicle control (Fig. 4B). This demonstrates that the higher abundance of acetylcarnitine and acylcarnitines in slow-twitch oxidative myofibers was due to increased FAO flux. To broadly analyze the myriad lipid species enriched in slow-twitch oxidative myofibers, relative to fast-twitch myofibers, MSEA^Lipid^ was performed on the LC-MS and MSI data. MSEA^Lipid^ confirmed that fatty acid esters, including acylcarnitines, were significantly enriched in slow-twitch myofibers both in LC-MS and MSI data (Fig. 4, C and D). Unexpectedly, sphingolipids were also enriched in slow-twitch oxidative myofibers, suggesting that increased lipid uptake is also a hallmark of slow-twitch oxidative myofibers (Fig. 4, C and D).

Given the enrichment in certain lipids and the need to provide sufficient free fatty acids to fuel FAO in myofibers when needed, we hypothesized that lipid droplets might exist in both slow- and fast-twitch myofibers. To prove this hypothesis, we used the BODIPY dye to specifically stain for lipid droplets in different subtypes of myofibers. Lipid droplets were similarly abundant in type 1 and type 2a oxidative myofibers, with a relatively uniform distribution along the myofiber (Fig. 4E). In contrast, the type 2b glycolytic myofibers were less enriched for lipid droplets, relative to the oxidative myofibers, except for a few type 2b myofibers that showed nonuniform, localized clustering of lipid droplets within the myofiber (Fig. 4F). Overall, our results support the conclusion that increased lipid uptake and FAO are defining metabolic traits of oxidative myofibers.

### MSI revealed that type 2b glycolytic myofibers harbor an oxidative subtype

**Fig. 5.**
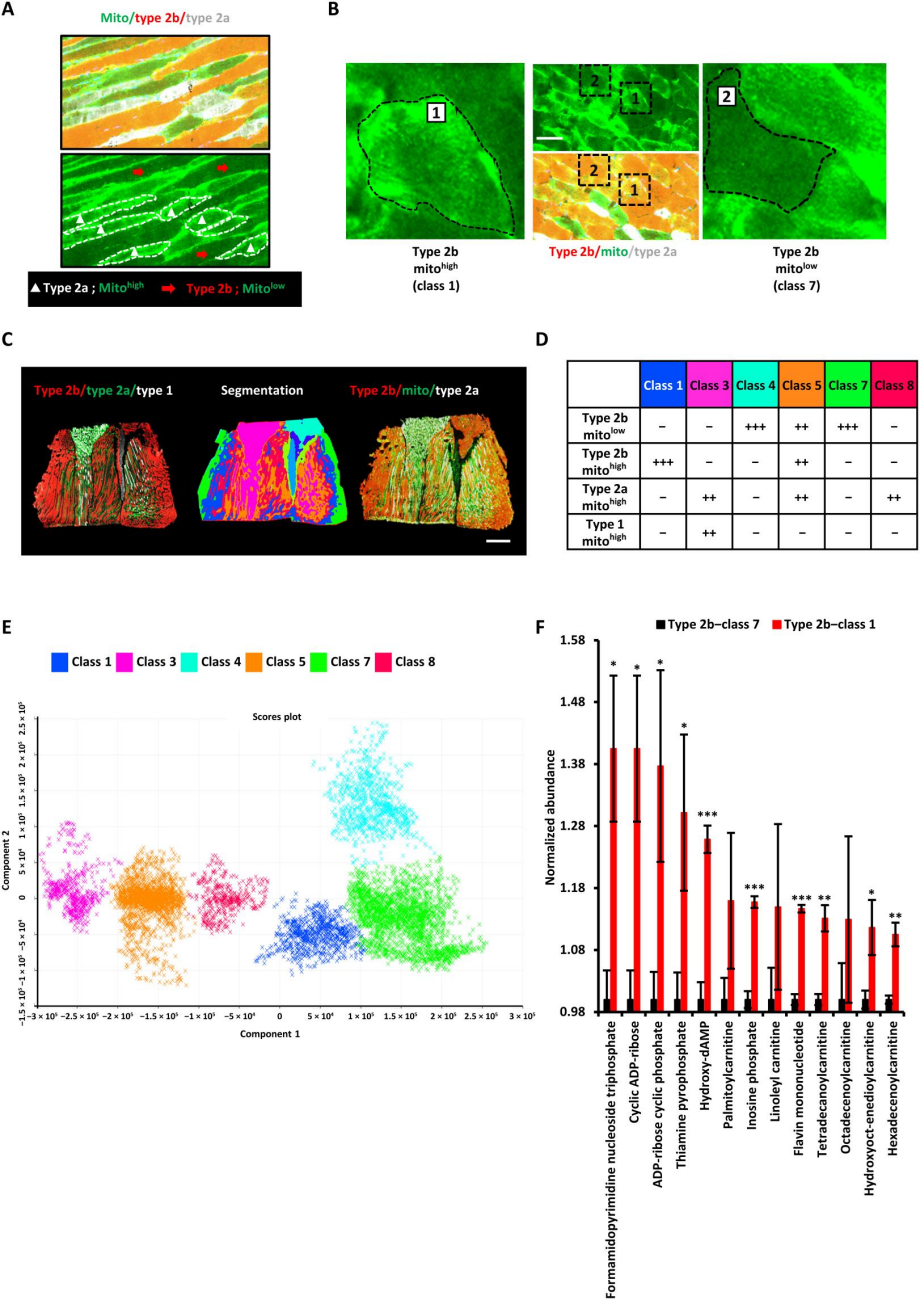
MSI reveals type 2b myofibers harbor an oxidative subtype. (**A**) Immunofluorescence staining of longitudinal sections for different myofiber subtypes and mitochondria (cytochrome c). White, type 1; red, type 2a; green, mitochondria (cytochrome c). (**B**) Immunofluorescence staining of transverse sections for different type 2 myofibers and mitochondria (cytochrome c). White, type 2a; red, type 2b; green, mitochondria (cytochrome c). (**C**) Three consecutive longitudinal sections of mouse GAS-SOL muscle were used for immunofluorescence staining and co-registered MSI. Left: Type 2b (red), type 2a (green), and type 1 (white). Middle: Unsupervised segmentation of the multidimensional MSI data into eight classes. Right: Type 2b (red), type 2a (white), and mitochondrial cytochrome c (green). Scale bar, 1 mm. (**D**) Scoring of the relative numbers of each myofiber subtype in each MSI metabolic class. Mito^high^, high abundance of mitochondrial cytochrome c; Mito^low^, lower abundance of mitochondrial cytochrome c. The more “+” signs, the higher the number of that subtype of myofibers. (**E**) PCA of the six MSI metabolite classes that corresponded to muscle regions. Three remaining MSI metabolite classes that corresponded to nonmuscle regions were omitted to avoid confounding the analysis (*N* = 3 mice’ GAS-SOL muscles; a total of 1269 MSI ion peaks were used for PCA). (**F**) Representative metabolites that were most up-regulated in type 2b mito^high^ (class 1) myofibers [means ± SEM; *N* = 3; *P < 0.05, **P < 0.01, and ***P < 0.001 compared with type 2b mito^low^ (class 7) myofibers; Student’s *t* test].

Some of the glycolytic myofibers' nonuniform enrichment of lipid droplets, more frequently associated with oxidative myofibers, led us to suspect that mitochondria and oxidative metabolism might also be enriched among some glycolytic type 2b myofibers. Immunofluorescent staining of cytochrome c, a mitochondrial heme protein specifically associated with the mitochondrial inner membrane critical for OxPhos, confirmed that gastrocnemius type 2a oxidative myofibers have a high abundance of mitochondria (Fig. 5A). In contrast, type 2b myofibers were largely devoid of mitochondrial cytochrome c, except for certain regions (Fig. 5B). These results not only indicated that mitochondrial cytochrome c staining was consistent with the well-established notion that oxidative myofibers have more mitochondria, while glycolytic (type 2b) myofibers have less mitochondria in general, but also suggested that at least some type 2b myofibers might have more mitochondria than expected.

To validate this suggestive finding, we returned to our MSI data and attempted to perform clustering of the spatial metabolomics data to see whether there are new hitherto uncharacterized metabolic subtypes of myofibers. After co-registering segmentation results with immunofluorescence staining images, among the nine different MSI regions identified by this spatial metabolomics pipeline (fig. S4, A and B), six were confirmed to be myofiber regions when compared with immunofluorescence images (Fig. 5, C and D), while the three filtered regions were either nonmuscle tissue regions or nontissue artifacts from cryosectioning. Some fingerprint metabolites can partially represent each of the MSI regions (fig. S4C). Multidimensional (or multimetabolite) information was more accurate in characterizing myofiber subtypes, as shown by PCA of the six myofiber MSI regions’ metabolomic spectra which clearly showed a progressive trend from class 3 → class 5 → class 8 → class 1 → class 7 → class 4 (Fig. 5E), suggesting a well-demarcated progression from type 1/2a oxidative myofiber subtypes to type 2b glycolytic myofiber subtypes.

When we carefully analyzed the distribution of mitochondrial cytochrome c in the type 2b glycolytic myofibers, we found that some type 2b myofibers (MSI class 1) have more mitochondria (mito^high^) than other type 2b myofibers (mito^low^, MSI class 4 and 7; Fig. 5D). Quantification of the results further revealed that MSI class 3 was mostly type 1 and type 2a slow-twitch oxidative myofibers, whereas MSI class 8 was mostly type 2a myofibers. MSI class 5 is a complex region consisting of three myofiber types: type 2a, type 2b mito^high^, and type 2b mito^low^ (Fig. 5D). This segmentation analysis was repeated with other skeletal muscle sections from other mice (*N* = 3), with similar conclusions. These unexpected results suggested that there may be more metabolic subtypes of myofibers than previously thought and that there is at least one new class of type 2b mito^high^ fibers among type 2b glycolytic myofibers.

To explore in detail the metabolic pathways and metabolites that distinguish type 2b mito^low^ and type 2b mito^high^ myofibers, class 7 and class 1 region-specific mass spectra were extracted for metabolomic analysis. The MSEA^Lipid^ results showed that type 2b mito^low^ myofibers were significantly enriched in triradylcglycerols and triacylglycerols (fig. S4D), whereas type 2b mito^high^ myofibers were significantly enriched in acylcarnitines (Fig. 5F and fig. S4E), just like slow-twitch oxidative myofibers (Fig. 4, C and D). In particular, the 13 metabolites that were significantly increased in type 2b mito^high^ myofibers, compared to type 2b mito^low^ myofibers, included six acylcarnitines, cyclic ADP-ribose, and thiamine pyrophosphate. Thiamine pyrophosphate is a diphosphate form of thiamine (vitamin B1), which serves as a cofactor for enzymes involved in the TCA cycle. Cyclic ADP-ribose is a second messenger involved in mobilizing intracellular Ca^2+^ release through ryanodine receptors on the sarcoplasmic reticulum and nucleus upon stimulation, e.g. in response to acetylcholine or β-adrenergic receptor agonists (*32*, *33*). Thus, type 2b mito^high^ myofibers resemble oxidative myofibers and might be related to neural or neuroendocrine stimulation of fast-twitch type 2b myofibers.

### Spatial transcriptomics revealed type 2b mito^high^ myofibers have unique extraocular muscle–like features

**Fig. 6.**
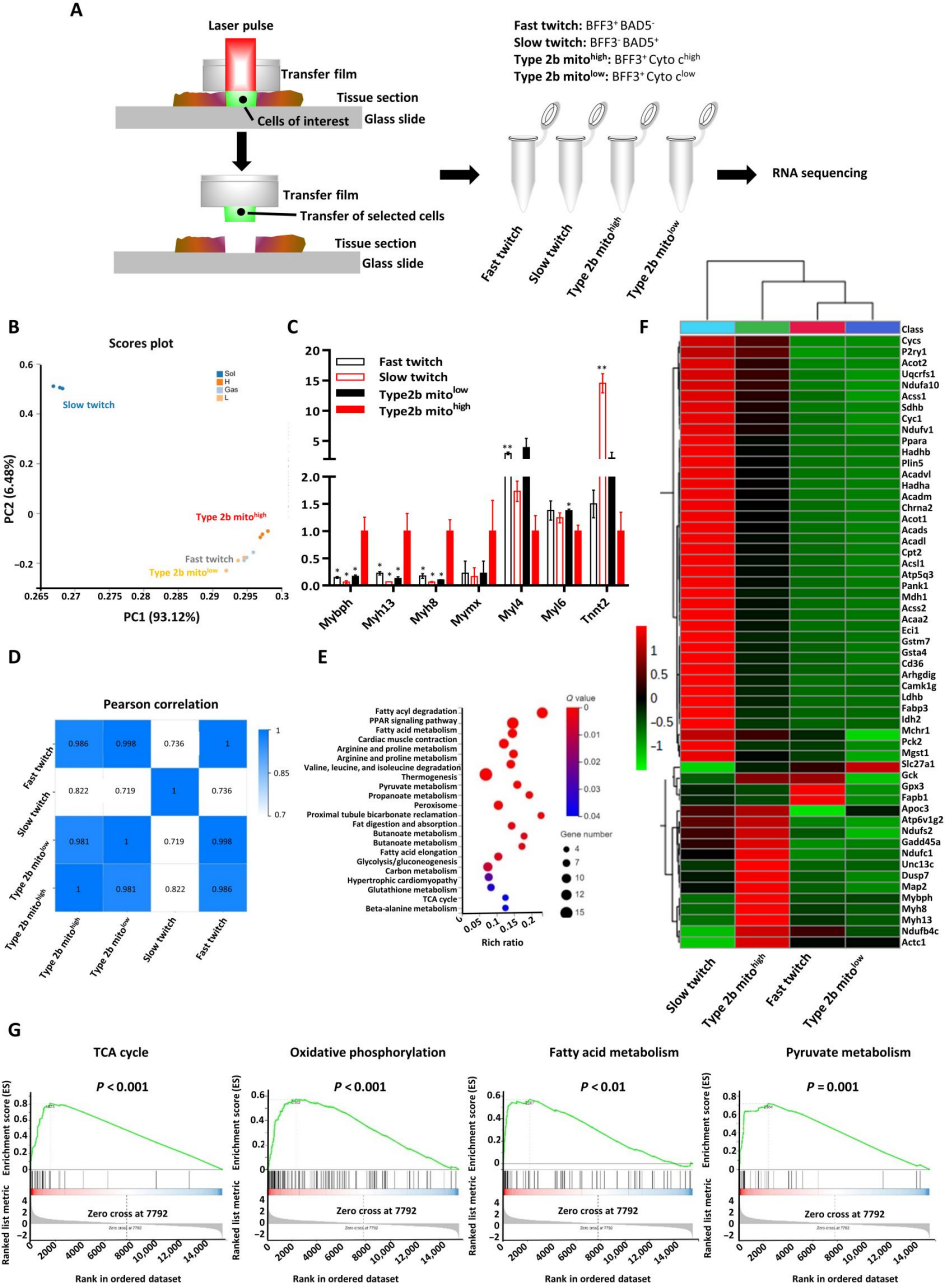
Spatial transcriptomics revealed type 2b mito^high^ myofibers have unique EOM-like features. (**A**) LCM workflow for RNA-seq of myofibers. For details, see Materials and Methods. (**B**) PCA of the type 2b mito^low^, type 2b mito^high^, fast-twitch, and slow-twitch myofibers (8 to 16 myofibers from one to two slides per sample, *N* = 3 samples per group). The two PCs explaining the largest parts of transcriptomic data variation are shown. (**C**) Transcript abundance of seven discriminative genes in PC1. FPKM indicates fragments per kilobase of transcript per million fragments mapped in RNA-seq (means ± SEM, *P < 0.05, **P<0.01, compared to type 2b mito^high^; Student’s *t* test). (**D**) Pearson correlation analysis between all myofibers’ RNA samples. (**E**) Bubble chart of Kyoto Encyclopedia of Genes and Genomes pathway enrichment analysis of DEGs in the type 2b mito^high^ myofibers, relative to type 2b mito^low^ myofibers. The *Q* value is the corrected Fisher’s exact test *P* value. Rich ratio refers to the ratio of the number of differential genes enriched in the pathway to the number of annotated genes. The greater the rich ratio, the greater the degree of enrichment. (**F**) Heatmap of top DEGs in the type 2b mito^high^ myofibers, relative to type 2b mito^low^ myofibers. Cutoff |log_2_ (fold change)| > 1, *P* ≤ 0.05. FDR-adjusted *P* values from likelihood tests were calculated using edgeR. (**G**) GSEA of the transcriptome of type 2b mito^high^ myofibers, relative to type 2b mito^low^ myofibers. GSEA signatures for the TCA cycle, OxPhos, fatty acid metabolism, and pyruvate metabolism were significantly enriched in type 2b mito^high^ myofibers. Family-Wise Error Rate (FWER)-adjusted *P* values for the GSEA test are calculated by permutation of statistics based on the Kolmogorov-Smirnov test.

Laser capture microdissection (LCM) is a high-resolution technique used to isolate specific cells from their surrounding tissues, with the aid of a laser beam, under direct microscopic visualization. To profile the transcriptomes of specific myofibers, we performed RNA sequencing (RNA-seq) after LCM isolation of specific myofibers. Before microdissection, immunofluorescence staining of type 2b myofibers (BFF3), type 1 myofibers (BAD5), and cytochrome c was performed. Thus, the fast-twitch, slow-twitch, type 2b mito^high^, and type 2b mito^low^ myofibers were labeled as BFF3^+^ BAD5^−^, BFF3^−^BAD5^+^, BFF3^+^cyto c^high^, BFF3^+^cyto c^low^, respectively (Fig. 6A). Using consecutive immunofluorescence staining images as a spatial reference atlas, ROIs in fresh cryosections were identified and microdissected with laser beams (fig. S5A). To ensure that our spatial transcriptomics workflow produced RNA samples that accurately reflected that of the corresponding cell compartments, we first evaluated the expression of well-established fast- and slow-twitch myofiber markers with quantitative reverse transcription polymerase chain reaction (fig. S5B). Having established the quality of our RNA samples, we proceeded with single-cell–depth RNA-seq. The RNA-seq data showed that the fast-twitch, type 2 mito^high^, and type 2b mito^low^ myofibers all had high abundance of *Myh4*, *Casq1*, *Myl1*, *Tnnc2*, *Tnni2*, and *Tnnt3* expression, whereas slow-twitch myofibers had high abundance of *Myh7*, *Casq2*, *Myl3*, *Tnnc1*, *Tnni2*, and *Tnnt1* expression, as expected of fast- and slow-twitch myofibers (fig. S5C) (*12*). Next, we performed gene set enrichment analysis (GSEA) on the 1658 differentially expressed genes (DEGs) between fast- and slow-twitch myofibers. In concordance with the metabolomic results above, the TCA cycle, fatty acid metabolism, and OxPhos genes were significantly enriched in slow-twitch myofibers, whereas glycolysis, PPP, and insulin signaling were significantly enriched in fast-twitch myofibers (fig. S5, C and D). These results confirmed that LCM–RNA-seq can be used as a spatial transcriptomics method to profile and distinguish myofibers identified using spatial metabolomics and immunofluorescence.

To understand the identity of type 2b mito^high^ myofibers, we visualized the transcriptomic gene expression of type 2b mito^high^ myofibers relative to type 2b mito^low^ myofibers, fast-twitch, and slow-twitch myofibers using PCA. Type 2b mito^high^ myofibers were distinct from the type 2b mito^low^ and fast-twitch myofibers, which intermixed among each other in one cluster (Fig. 6B). Furthermore, the second component of the PCA (PC2) indicated that oxidative slow-twitch myofibers are closer to type 2b mito^high^ myofibers than type 2b mito^low^ myofibers, relative to fast-twitch myofibers (Fig. 6B). This finding was supported by correlation analysis among these four groups of myofibers (Fig. 6D) and was consistent with our mitochondrial staining and spatial metabolomic profiling of type 2b mito^high^ myofibers (Fig. 5). To further understand why type 2b mito^high^ myofibers are so uniquely different from the other myofibers along the first component of PCA (PC1), we identified some discriminative genes with high loading values in PC1. We found that *Mybph*, *Myh13*, *Myh8*, and *Mymx* genes were uniquely abundant, whereas *Myl4*, *Myl6*, and *Tnnt2* genes were uniquely low in type 2b mito^high^ myofibers, relative to other myofibers (Fig. 6C). The *Myh13* gene encodes the superfast myosin heavy chain (previously) only found in extraocular muscles (EOMs) and laryngeal muscles; the *Myh8* gene encodes a fetal myosin heavy chain, while the *Mymx* gene encodes the proregenerative fusogen myomixer. These features are reminiscent of EOMs, which can undertake fatigue-resistant yet superfast-twitch contractions and also express high levels of extraocular myosin, embryonic and type 2 myosins, regenerative markers, and mitochondrial genes (*34*–*36*). Thus, we hypothesized that type 2b mito^high^ myofibers are myofibers in the mouse hindlimb that resemble fatigue-resistant, superfast-twitch EOMs.

To confirm that the type 2b mito^high^ myofibers have an EOM-like myofiber signature, we first used a cutoff *P* ≤ 0.01 and False Discovery Rate (FDR) ≤ 0.05 to discover 25 genes differentially expressed in type 2b mito^high^ myofibers, compared to type 2b mito^low^, fast-twitch, and slow-twitch myofibers. The up-regulated genes in type 2b mito^high^ myofibers included a sarcomeric and neuromuscular gene signature known to be highly enriched in EOMs, e.g., *Myh13*, *Myh8*, *Myh1*, *Actc1*, *Map2*, and *Chrna2* (*34*, *37*). To further investigate the characteristics of type 2b mito^high^ myofibers, relative to type 2b mito^low^ myofibers, we used the cutoff values of |log_2_foldchange| > 1, *P* ≤ 0.05, and FDR ≤ 0.05 and discovered 337 genes. Kyoto Encyclopedia of Genes and Genomes (KEGG) pathway analysis of these genes showed that 22 pathways were significantly enriched in type 2b mito^high^ myofibers, including peroxisome proliferator–activated receptor (PPAR) signaling, fatty acid degradation, fatty acid metabolism, pyruvate metabolism, and the TCA cycle, whereas only arginine and proline metabolism was significantly enriched in type 2b mito^low^ myofibers (Fig. 6E). Mitochondrial FAO and OxPhos-related genes such as *Acaa2*, *Acadl*, *Acadm*, *Acads*, *Acadvl*, *Acot1*, *Acot2*, *Acsl1*, *Acss1*, *Acss2*, *Eci1*, *Hadha*, *Hadhb*, *Pank1*, *Pck2*, *Idh2*, *Mdh1*, *Sdhb*, *Atp5g3*, *Atp6v1g2*, *Ndufs2*, *Ndufc1*, *Ndufb4c*, *Ndufa10*, *Ndufv1*, *Cyc1*, *Cycs*, *Uqcrfs1* and PPAR signaling target genes such as *Apoc3*, *Slc27a1*, *Cd36*, *Ppara*, *Fabp3*, and *Plin5* were all significantly higher in type 2b mito^high^ myofibers, compared to type 2b mito^low^ myofibers (Fig. 6, F and G), consistent with the fatigue-resistant metabolism of EOMs (*34*, *37*). Some glycolysis genes (*Ldhb* and *Gck*) were also further enriched in type 2b mito^high^ myofibers, consistent with the superfast-twitch features of EOMs (*34*, *37*). However, type 2b mito^high^ myofibers also differ from conventional EOMs, in having higher expression of *Mybph* (myosin binding protein H) and lower expression of *Myl4* (embryonic/atrial myosin light chain 1) and *Tnnt2* (cardiac troponin T) (Fig. 6C) than other hindlimb myofibers (*34*, *37*). In conclusion, the LCM–RNA-seq profiling of type 2b mito^high^ myofibers in the hindlimb suggests that they are very similar but not completely the same as superfast EOMs.

## DISCUSSION

Skeletal muscle composition is heterogeneous because each myofiber type has specific metabolic properties. Understanding the dynamic metabolic states of myofibers is helpful to understand the mechanism of myofiber remodeling. MSI is a powerful tool for studying biological phenomena due to its ability to map thousands of molecules without any labeling and in only one mass spectrum acquisition sequence. However, high–spatial resolution myofiber MS images for metabolomic profiling are hindered by the inaccurate annotation, inaccurate co-registration, and poor spatial resolution of MS images. In this study, we demonstrated the metabolomic profiling of slow- and fast-twitch myofibers with MSI after cross-validation by LC-MS–based metabolomic analysis. MSI and LC-MS, in combination, produced spatial metabolomics results that were more reliable. Furthermore, we achieved high–spatial resolution myofiber MS images through longitudinal muscle cryosectioning, thereby increasing each myofiber's cross-sectional area and, thus, ion abundance. As a result, we discovered a new subtype of type 2b mito^high^ myofibers. The present work provides a new strategy for high–spatial resolution metabolomics and illustrates its applicability in identifying known and previously unidentified subtypes of myofiber cells.

MSI allows direct investigations of the spatial distribution of metabolites in biological tissues, but the analytical depth of MSI is limited. In the present study, the mitochondria-related metabolic pathways, e.g., FAO, TCA cycle, and OxPhos (*38*), are highly enriched in slow-twitch myofibers according to the LC-MS data. However, only the intermediates of FAO, e.g., acetylcarnitine and acylcarnitines, were detected by MSI in slow-twitch myofibers. Free fatty acids and TCA cycle organic acids are carboxyl-containing metabolites that are difficult to detect by MSI without on-tissue derivatization (*39*). Acetylcarnitine and acylcarnitines are important intermediates in FAO, an important energy source for mammalian cells, and previous studies have also demonstrated the spatial distributions of some acylcarnitines in various tissues, e.g., cancer tissues (*40*), skeletal muscles (*20*), and pancreatic tissue (*41*). Venn diagram analysis showed that carnitine, acetylcarnitine, butenylcarnitine, hexadecenoylcarnitine, hydroxypentadecanoylcarnitine, linoleylcarnitine, oleoylcarnitine, palmitoylcarnitine, tetradecanoylcarnitine, and tetradecenoylcarnitine were detected as differential metabolites in both LC-MS and MSI. In addition, a large variety of acylcarnitines, including dodecanoylcarnitine, hexanoylcarnitine, and propionylcarnitine, were found to be significantly enriched in slow-twitch myofibers by LC-MS analysis but not in MSI analysis, possibly due to the higher sensitivity of LC-MS. Purine and pyrimidine metabolism were significantly enriched in fast-twitch myofibers by both LC-MS and MSI analysis, including AMP, deoxyinosine monophosphate, 7-methylguanosine 5-monophosphate, ADP-ribose phosphate, deoxyuridine triphosphate, and uridine diphosphate, consistent with a previous study that also reported that IMP and ADP are enriched in the glycolytic myofibers. The differential abundance in nucleobases between fast- and slow-twitch myofibers could be correlated with the need to power the rapid myokinase reaction and the slower nucleotide salvage rate in fast-twitch myofibers (*20*, *42*, *43*).

We also applied the spatial denoising methods and the segmentation pipeline algorithms to cluster the MALDI-MSI spatial spectra and uncovered a new metabolic subtype of type 2b mito^high^ myofibers. Moreover, we used LCM–RNA-seq to define the gene expression profile of these rare type 2b mito^high^ myofibers as *Myh13*^+^ superfast myofibers in the hindlimb for the first time. Several other lines of research had also suggested the possibility of new myofiber subtypes under physiological and pathological conditions, but none of them had provided definitive evidence. For example, a few studies have revealed heterogeneity in myosin subtype composition along the longitudinal fiber axis in some limb muscles (*44*) and EOMs (*45*). This heterogeneity is thought to be a common feature in these muscles to confer the ability to control delicate skeletal muscle movements. In addition, it was found that the mitochondrial component of the glycerol-3-phosphate shuttle can be found in some type 2b myofibers (*44*, *46*), but the underlying reasons had remained an unexplained mystery so far. Given that each EOM myofiber is multiply innervated and that EOM-specific Myh13 is expressed in myonuclear domains near neuromuscular junctions in rabbit EOMs (*35*), future work could explore whether the enrichment of EOM-like genes, metabolites, and Ca^2+^-mobilizing cyclic ADP-ribose in type 2b mito^high^ myofibers is due to enhanced neural or neuroendocrine stimulation of limb type 2b myofibers, leading to EOM-like fatigue-resistant metabolism and superfast-twitch genetic programs. This is plausible because EOM-like superfast muscles can also be found in the wing muscles of hummingbirds (*34*). Thus, more metabolic subtypes of myofibers and other cell types might be awaiting discovery by combining high-resolution spatial metabolomics with spatial transcriptomics. In conclusion, using MSI to visualize myofibers can improve our understanding of myofiber remodeling under physiological and pathological conditions. Our approach has the potential to provide critical insights into the metabolic regulation of fundamental biological processes and disease states.

## MATERIALS AND METHODS

### Chemical and reagents

DHB was obtained from ProteoChem (Hurricane, HT, USA). 1,5-DAN, formic acid, and trifluoroacetic acid (TFA) were purchased from Sigma-Aldrich (St. Louis, MO, USA). High-performance LC–grade water (H_2_O), acetone, methanol, and acetonitrile (ACN) were purchased from Fisher Scientific (Fair Lawn, NJ). BAD5, BFF3, BF35, and SC71 antibodies were purchased as 1-ml supernatants from the Developmental Studies Hybridoma Bank at the University of Iowa (Iowa City, IA); cytochrome c antibody was purchased from Abcam (Cambridge, England). Secondary antibodies Alexa Fluor 488 Goat anti-Mouse IgG1, Alexa Fluor 568 Goat anti-Mouse IgM, and Alexa Fluor 647 Goat anti-Mouse Ig2b were purchased from Life Technologies (Grand Island, NY). 4′,6-Diamidino-2-phenylindole (DAPI) was purchased from Beyotime Biotechnology (Shanghai, China).

### Sample preparation

All animal experiment procedures were approved by the Institutional Animal Care and Use Committee of the Institute of Zoology (Chinese Academy of Sciences, IOZ-IACUC-2022-170). All the mice used in this study were of a C57BL/6J background. Both sexes were used throughout the study, and no animals were excluded from analysis. Mice were randomly assigned to experimental groups. All experimental groups were organized such that they were litter- and sex-matched. After mice were kept under isoflurane for 30 s and sacrificed, the EDL, GAS-SOL, and SOL were quickly removed from the mouse body and immediately frozen in liquid nitrogen and stored at −80°C. The EDL and SOL muscles were subjected to a metabolite extraction procedure modified from previously published protocols (*47*). Samples were weighed and then homogenized with a TissuePrep 24 homogenizer (Gering, Beijing, China) in 80% (v/v) methanol, which was precooled to −80°C. The homogenates were centrifuged at 5000*g* for 15 min, and the resulting supernatants were collected for LC-MS analysis.

The frozen GAS-SOL muscles were cryosectioned at 10-μm thickness on a Leica CM3050s cryostat (Leica Microsystems, Wetzlar, Germany) at −20°C, thaw-mounted onto indium tin oxide–coated glass slides (Bruker Daltonics, Bremen, Germany), and stored at −80°C before analysis. On the day of analysis, samples were brought to room temperature in a vacuum pump. After drying in vacuum for 10 min, matrix solution was applied to tissue sections by a robotic sprayer (TM-Spray, HTX Technologies). DHB and 1,5-DAN were used with the positive ion mode of MALDI-fourier-transform ion cyclotron resonance (FTICR) and AP-MALDI-quadrupole time-of-flight (QTOF), respectively. Parameters of the spraying method were as follows: Nozzle nitrogen gas pressure, velocity, and track spacing were set to 10 psi, 1200 mm/min, and 2.5 mm. The flow rate was 0.125 ml/min, and 14 cycles of DHB (15 mg/ml) in 90% ACN containing 0.1% TFA while 0.12 ml/min and 4 cycles of 1,5-DAN (10 mg/ml) in 70% ACN containing 0.1% TFA were sprayed on tissue surfaces. After matrix application, the slides were put in a slide adapter for MSI analysis.

### Matrix-assisted laser desorption/ionization–mass spectrometry imaging

MALDI-MSI experiments were performed on a MALDI-FTICR MS instrument (SolariX 9.4T, Bruker Daltonics) equipped with a SmartBeam II laser source (2-kHz Nd: YAG×3 laser at 355 nm; Bruker Daltonics). Before analysis, the method was calibrated with DHB and sodium trifluoroacetate. All images were collected using the minimum laser setting with a pixel spacing of 50 or 25 μm in both *x* and *y* dimensions. Data were collected in positive ion mode from *m*/*z* 100 to 1000 with 400 laser shots averaged per pixel, and the laser is fired at a repetition rate of 1000 Hz. The device parameters for MALDI-MSI were chosen as follows: plate offset voltage, 100 V; deflector plate voltage, 180 V; collision voltage, −10 V; DC extract bias, 0.8 V; collision radio frequency amplitude, 1000 Vpp; and time of flight, 0.7 ms. All data were processed using the FlexImaging 3.0 software and SCiLS Lab software (Bruker Daltonics, Germany). High-resolution MS spectra were used to distinguish different compounds, and the identification of compounds was achieved by precisely matching *m*/*z* with the HMDB (HMDB 5.0, www.hmdb.ca) and METLIN database (https://metlin.scripps.edu). The mass error tolerance was set at 10 parts per million (ppm).

### AP–MALDI-MSI

An AP-MALDI ion source [AP/MALDI (ng) UHR source, MassTech Inc., Columbia, MD] coupled to an AB SCIEX TripleTOF 5600+ system was used. Analyst software (AB SCIEX LLC, Framingham, MA) was used to control the MS parameters. Laser energy of 30% and 5-kV voltage were applied onto the plate, and capillary temperature of 200°C was used. For MSI experiments, constant speed raster motion was used with 60-μm spatial resolution and plate velocity dependent on scan time. Acquisition was performed in full scan with positive and negative polarity and a mass range of *m*/*z* 100 to 1000. Acquired raw data were converted from the proprietary raw format to mzML using ProteoWizard followed by conversion to imzML format using the imzMLConverter. MSiReader software (*48*) was used to create peak lists and generate images from the data. High-resolution MS spectra were used to distinguish different compounds, and the identification of compounds was achieved by precisely matching *m*/*z* with the HMDB (HMDB 5.0, www.hmdb.ca) and METLIN database (https://metlin.scripps.edu). The mass error tolerance was set at 10 ppm.

### On-tissue MS/MS (MS2) analysis

On-tissue MS/MS analysis was performed on the Triple TOF 5600+ (AB SCIEX) equipped with an AP-MALDI source (MassTech, Columbia, MD). The positive ion mode was used to provide sufficient ions for validating the presence of each potential compound, as well as product/fragment ions for structural characterization. The conditions of TOF-MS scan types were as follows: TOF masses range was set at *m*/*z* 750 to 850; accumulation time, 0.25 s; ion source gas 1, 0 psi; ion source gas 2, 0 psi; curtain gas, 10 psi; temperature, 220°C; declustering potential, 80 V; and collision energy, 10 eV; ion spray voltage floating was set at 3000 V. For product/fragment ion scan type, the parameters were almost the same except that the TOF masses range was set at *m*/*z* 50 to 1000; accumulation time was set at 0.1 s, and collision voltage was set at 35 ± 15 eV. All operations and acquisitions were controlled by Analyst TF1.6 software (AB SCIEX). MS/MS data were analyzed with the PeakView software v.1.2 (AB SCIEX). XIC manager and formula finder were used in data processing. All metabolites were annotated by matching MS/MS spectra to the HMDB (HMDB 5.0, www.hmdb.ca) and METLIN database (https://metlin.scripps.edu).

### LC-MS analysis

The EDL and SOL muscles were used for metabolite extraction. ACQUITY I-class ultra-performance LC (UPLC) system (Waters) coupled in line with Xevo G2-XS QTOF hybrid mass spectrometer (Waters) was used for intracellular metabolite profiling. The separation was performed using a reversed-phase column (ACQUITY UPLC HSS T3 Column; 2.1 × 100 mm in length; 1.7-μm particle size), with the following solvents: mobile phase A (100% water) and B (100% ACN) both containing 0.1% formic acid, and the following elution gradient was used: 0-min 95% A, 8.5-min 50% A, 12-min 2% A, and 16-min 95% A. The flow rate was 0.4 ml/min. The injection volume was 2 μl.

The mass spectrometer was used in positive and negative electrospray ionization mode for data acquisition using UPLC/MS^E^, which allowed both precursor and product/fragment ion data to be acquired in one run. Typical source conditions for maximum intensity of precursor ions were as follows: capillary voltage, 2.5 kV in positive mode and 2.0 kV in negative mode; sample cone, 40 V; source temperature, 120°C; desolvation temperature, 20°C; cone gas flow rate, 30 liters/hour; and desolvation gas (N_2_) flow rate, 800 liters/hours. All analyses were performed using the lockspray, which ensured accuracy and reproducibility. Leucine-enkephalin (5 ng/ml) was used as the lockmass generating a reference ion (positive mode: *m*/*z* 556.2771; negative mode: *m*/*z* 554.2615) and introduced by a lockspray at 10 μl/min for accurate mass acquisition. MS data processing was conducted using the Progenesis QI software (Nonlinear Dynamics, Waters). Raw data were imported into Progenesis QI for peak alignment and peak picking, and automatic identification of metabolites was performed by searching HMDB and METLIN databases based on the accurate *m*/*z* matching of high-resolution MS^E^ data.

### Immunofluorescence staining

Muscle sections were fixed in environmentally friendly GD solution (G1111, Servicebio, China) for 15 min; washed 3 × 5 min in phosphate-buffered saline (PBS); blocked for 60 min at room temperature in PBS containing 2% bovine serum albumin, 5% goat serum, and 0.2% Triton X-100; and subsequently incubated in primary antibodies overnight at 4°C. Secondary antibody detection was performed for 1 hour at room temperature. Nuclei were labeled with DAPI. Images were taken with confocal microscopy (Nikon A1R, Nikon Instruments Inc., Tokyo, Japan).

### Myofiber LCM and RNA-seq

Consecutive sections of gastrocnemius muscle were cryosectioned at 10-μm thickness on a Leica CM3050s cryostat (Leica Microsystems, Wetzlar, Germany) at −20°C and thaw-mounted onto Molecular Machines & Industries (MMI) membrane slides with RNAse free (MMI, 50102). All sections were laser microdissected before RNA purification; the slow-twitch, fast-twitch, type 2b mito^high^, and type 2b mito^low^ myofibers (8 to 16 myofibers per sample, *N* = 3 samples per myofiber group) were identified on the basis of BAD5 (type 1), BFF3 (type 2b), and cytochrome c (mitochondria) immunofluorescence staining of parallel sections. Laser microdissection was performed with a MMI Cellcut LMD system (MMI GmbH, Eching, Germany) coupled with an Eclipse TE-2000 fluorescent microscope (Nikon Instruments, Melville, NY, USA). Selected areas identified by immunofluorescence staining were cut from the tissues using an ultraviolet laser beam. RNA extraction was performed using the RNase micro Kit (QIAGEN, Copenhagen, Denmark). mRNA amplification and cDNA reverse transcription were performed using the Single Cell Full Length mRNA-Amplification kit (Vazyme, #N712), and sequencing libraries were constructed using the TruePrep DNA library Prep Kit V2 for Illumina (Vazyme, #TD503). Before library construction, RNA integrity values, 28*S*/18*S*, and the fragment length distribution and molar concentrations were analyzed using the Invitrogen Qubit (Thermo Fisher Scientific) and a 2100 Bioanalyzer (Agilent Technologies) and evaluated in the 2100 Expert software (Agilent Technologies). PE100 sequencing was performed on the HiSeq 4000 platform (BGI), generating about 11.06G Gb of data per sample. Postsequencing low-quality reads, reads with adaptors, and reads with unknown bases were filtered from the raw data to obtain clean data. Clean reads were mapped to the mouse GRCm38.p6 reference genome using HISAT algorithm and to the reference transcriptome using Bowtie2 software. The average mapping ratio was 90.94%, and a total of 16,386 genes were identified. Expression levels of genes were calculated by the fragments per kilobase of transcript per million fragments mapped method using the RSEM software package, and differential gene expression was analyzed using edgeR.

### Data analysis

SCiLS Lab software (Bruker Daltonics, Germany) was used for univariate statistical hypothesis test, PCA, ROC analysis, and unsupervised characterization of MSI data. MALDI-MSI raw data were first imported into the SCiLS Lab 2022a software and converted to the SCiLS Lab format. The standard segmentation pipeline starts with data preprocessing, including baseline removal, root mean square normalization, and peak picking. After peak picking, edge-preserving median filter denoising was applied to the *m*/*z* images of detected peaks. The reduced and processed spectra were thus used for Student's *t* test, PCA, ROC analysis, and spatial segmentation. The area under the ROC (AUC) was used to explore peaks that discriminated between fast- and slow-twitch myofibers. The AUC discrimination quality varied in the interval between 0.0 and 1.0. An AUC near 0.0 or 1.0 means good discrimination in one or the other class, while an AUC near 0.5 means poor discrimination. Spatial segmentation was achieved by using a semi-supervised *K*-means clustering algorithm; the results are presented as spatial segmentation maps and corresponding hierarchical dendrograms composed of clusters, with pseudo-colors assigned to pixels (or individual spectra) belonging to each cluster. We also used the MetaboAnalyst software (www.metaboanalyst.ca) for detailed metabolite analysis. The MSEA, MSEA^Lipid^, and Pathway analysis modules in MetaboAnalyst were used for metabolic pathways enrichment.

### Statistical analysis

The reproducibility of metabolites was evaluated by ICCs calculated in SPSS 24.0 using a two-way mixed model with consistency and average measures as reported (*49*, *50*), and the values were between 0 and 1. An ICC ≥ 0.75 was considered to represent excellent reproducibility, 0.60 to 0.75 to represent good reproducibility, and <0.4 to represent poor reproducibility. All data were presented as means ± SEM unless mentioned otherwise. Sample sizes (*n*) and statistical methods used to assess significant differences in each statistical analysis are noted in each figure legend.
