# Supplementary material for: Spatial metabolomics reveals skeletal myofiber subtypes
Source: Sci Adv. 2023 Feb 3;9(5):eadd0455. doi: 10.1126/sciadv.add0455 (PMC10939097; doi:10.1126/sciadv.add0455)
Supplement: Supplementary file 1 — Figs. S1 to S5 [file sciadv.add0455_sm.pdf]

Science Advances
AAAS

Supplementary Materials for

Spatial metabolomics reveals skeletal myofiber subtypes

Lanfang Luo *et al.*

Corresponding author: Ng Shyh-Chang, huangsq@ioz.ac.cn; Taoyan Liu, liutaoyan@ioz.ac.cn

Sci. Adv. **9**, eadd0455 (2023)
DOI: 10.1126/sciadv.add0455

The PDF file includes:

Figs. S1 to S5
Legends for tables S1 to S4

Other Supplementary Materials for this manuscript includes the following:

Tables S1 to S4

Figure S1. Spatial distribution of acylcarnitines in fast- and slow-twitch muscles.

204.1275 L-Acetylcarnitine | **370.2959 Cis-5-Tetradecenoylcarnitine** | **372.3115 Tetradecanoylcarnitine**

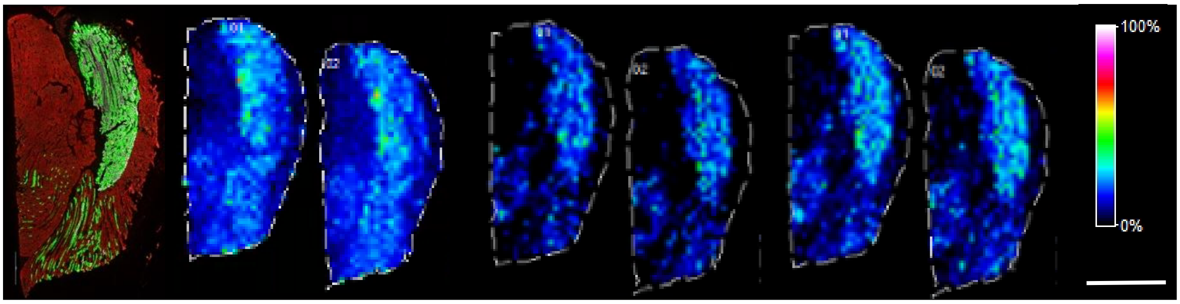

396.3118 9,12-Hexadecadienoylcarnitine | **398.3275 Trans-Hexadec-2-enoylcarnitine** | **400.3418 L-Palmitoylcarnitine**

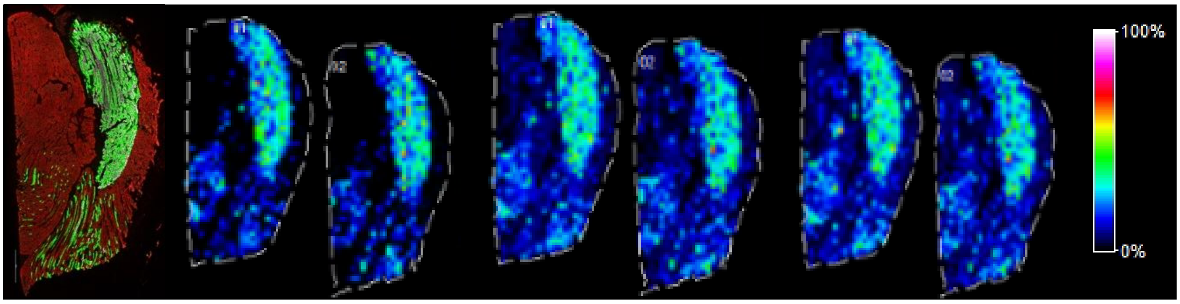

424.3431 Linoelaidylcarnitine | **426.3585 Octadecenoylcarnitine** | **428.37241 Stearoylcarnitine**

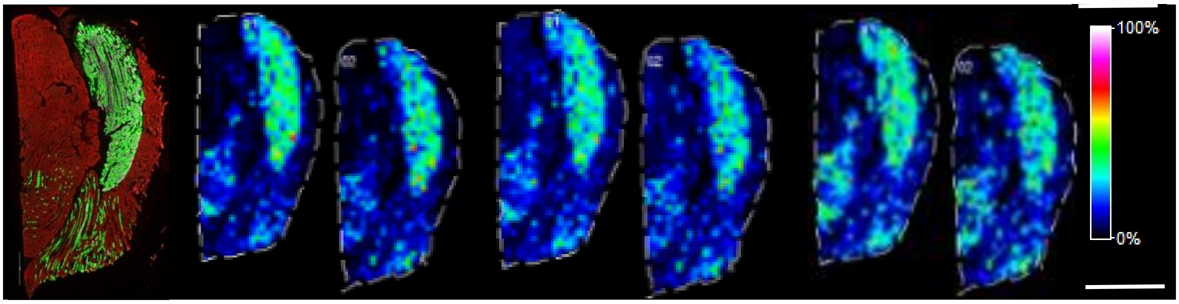

Figure S1. Spatial distribution of acylcarnitines in fast- and slow-twitch muscles.

Immunofluorescence staining and MSI analysis of the hindlimb's gastrocnemius and soleus muscles. Green, slow-twitch myofiber. Red, fast-twitch myofiber. Scale bar, 2.5mm.

Figure S2. On tissue MS/MS of three fingerprint metabolites with discriminative spatial distributions in the muscles.

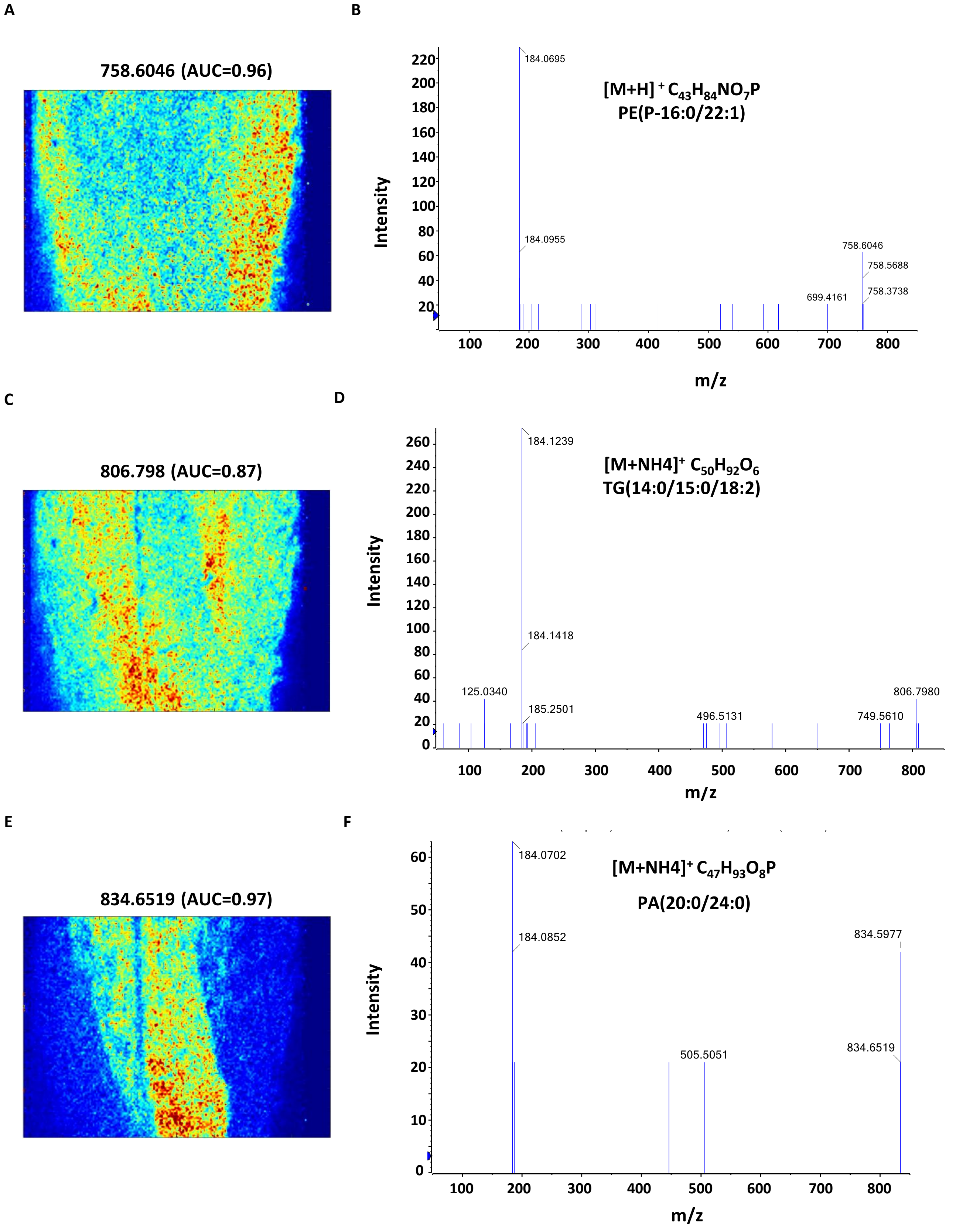

Figure S2. On tissue MS/MS of three fingerprint metabolites with discriminative spatial distributions in the muscles.

A, C, E. Mass spectrometry imaging of **(A)** 758.6046, **(C)** 806.798 and **(E)** 834.6519 in longitudinal cryosections of gastrocnemius-soleus (gas-sol) muscle. 758.6046: AUC=0.96, ICC=0.83, p-value<0.001, N=24; 806.798: AUC=0.87, ICC=0.92, p-value=0.009, N=24; 834.6519: AUC=1, ICC=0.97, p-value<0.001, N=24. AUC: the area under the receiver operating characteristic curve measures the discrimination quality. An AUC near 0.0 or 1.0 means a good discrimination with high intensities of spectra in one class, while an AUC near 0.5 means poor discrimination.

B, D, F. MS/MS product ion spectrum of **(B)** 758.6046, **(D)** 806.798 and **(F)** 834.6519 in longitudinal cryosections of gas-sol muscle. The formulae of 758.6046, 806.798 and 834.6519 were identified by the formula finder of the Peakview software based on accurate mass, isotope distribution and fragments. The formulae of 758.6046, 806.798 and 834.6519 are C43H84NO7P, C50H92O6, C47H93O8P, corresponding to their $[M+H]^+$, $[M+NH4]^+$ and $[M+NH4]^+$ ions, respectively. The metabolites identified by database searching and spectral matching for 758.6046, 806.798 and 834.6519 are PE (P-16:0/22:1), TG (14:0/15:0/18:2), and PA (20:0/24:0), respectively.

Figure S3. LC-MS quality control with PCA, and biomarker selection with PLS-DA.

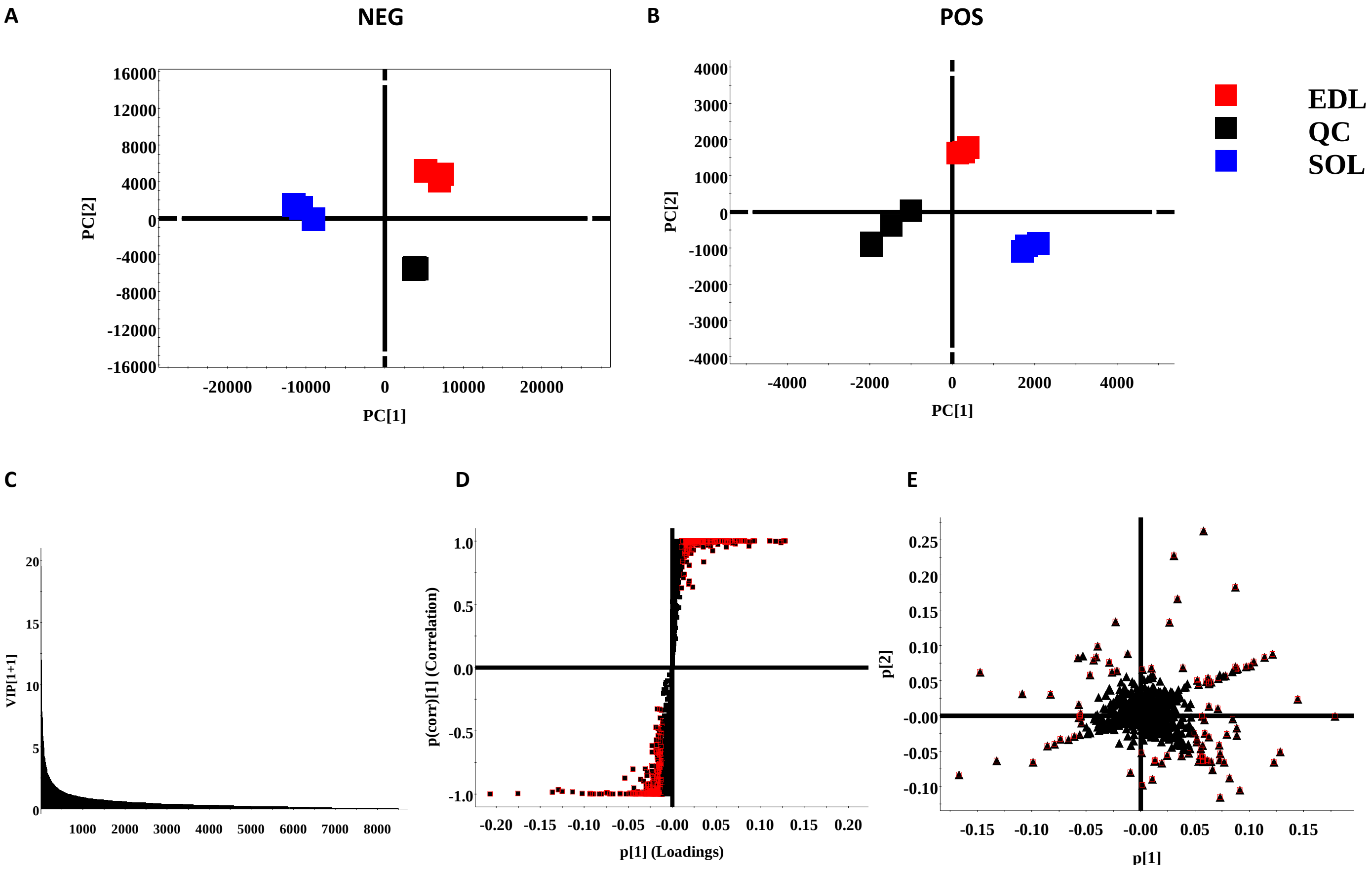

Figure S3. LC-MS quality control with PCA, and biomarker selection with PLS-DA.

A,B. Principal component analysis (PCA) shows the distributions of quality control (QC), EDL and SOL samples in **(A)** negative ion mode, and **(B)** positive ion mode.

C,D,E. Metabolite biomarkers in EDL and SOL (Table 3) according to the orthogonal partial least squares discriminant analysis (PLS-DA) regression model and the derived (C) Variable importance in the Project (VIP) values, (D) S-plot and (E) loading plot, analyzed by Progenesis QI and Ezinfo software. Cutoff loading scores ≥0.05 and VIP scores ≥1 were used. The S-plot visualizes the modeled covariance (X-axis) and modeled correlation (Y-axis) from the PLS-DA on a scatter plot, allowing for selection of metabolites as biomarkers. Metabolites located far from the origin represent importance in the model, i.e. the red dots have VIP scores ≥1. The X-axis and Y-axis of the loading plot represent the coefficient weights of the metabolites along the two predictive components derived from the PLS-DA model.

Figure S4. Segmentation analysis of high spatial resolution MSI.

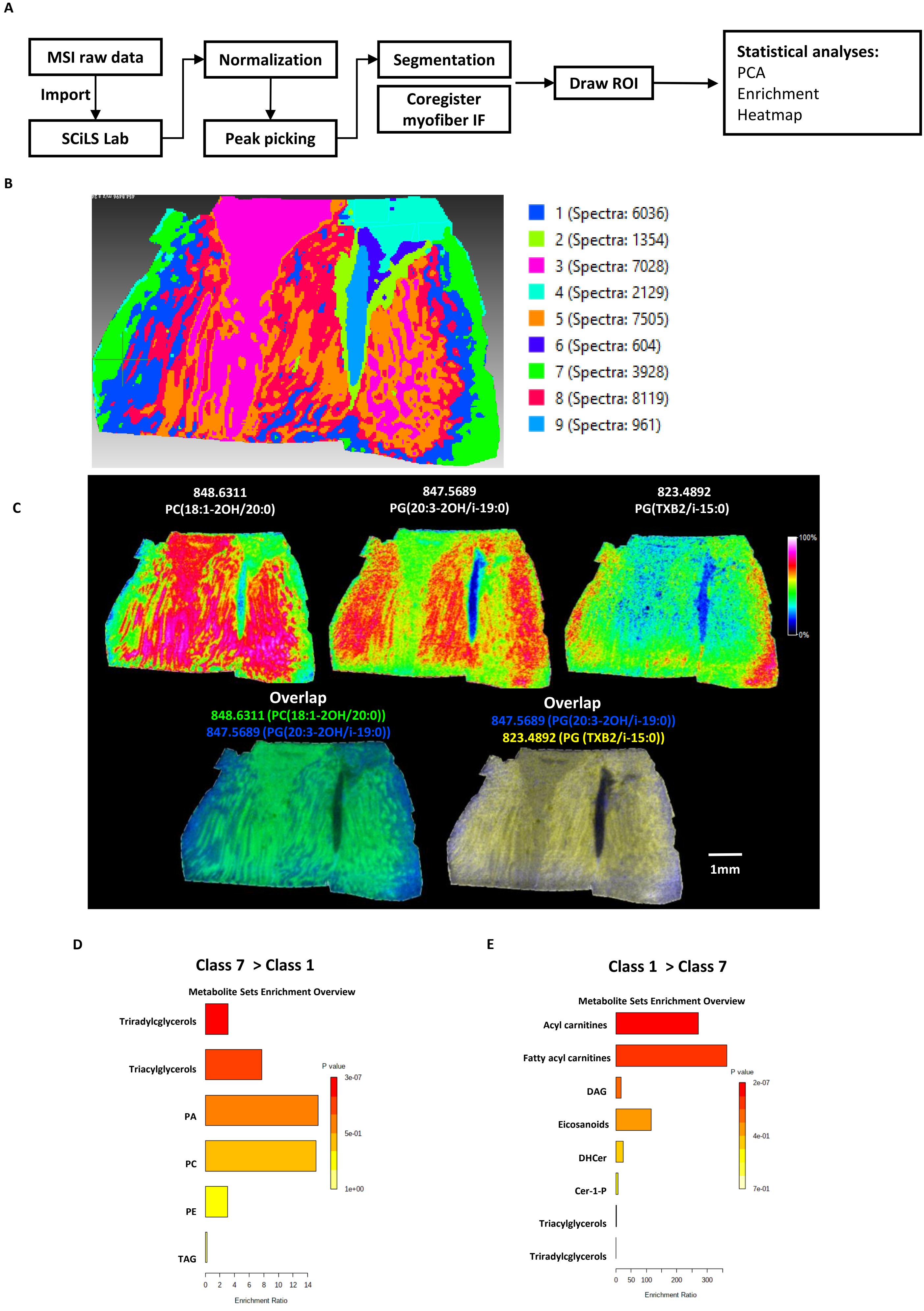

Figure S4. Segmentation analysis of high spatial resolution MSI.

A. Spatial metabolomic analysis workflow for muscle fiber MSI and immunofluorescence (IF) data. For details, see main text and Material and Methods section.

B. 9 clusters were obtained by segmentation analysis, and the number of m/z spectra that define each cluster is shown.

C. Overlap between MS images of oxidative and glycolytic myofiber fingerprint metabolites, where m/z 848.6311 can mark the type 1/2a oxidative (class 3) myofibers, m/z 847.5689 and m/z 823.4892 can mark the type 2b glycolytic (class 1 and 7) myofibers respectively. Scale bar, 1mm.

D,E. $MSEA^{Lipid}$ of differentially abundant lipids from type 2b $mito^{high}$ (class 1) and type 2b $mito^{low}$ (class 7). MSEA of significantly upregulated lipids in **(D)** type 2b $mito^{low}$ (class 7) myofibers (Triradylcglycerols: $p<0.001$; Triacylglycerols: $p<0.001$; PA: $p<0.001$; PC: $p<0.001$) and **(E)** type 2b $mito^{high}$ (class 1) myofibers (Acyl carnitines: $p<0.001$; Fatty acyl carnitines: $p<0.001$; DAG: $p=0.005$; Eicosanoids: $p=0.009$; DHCer: $p=0.004$). Bonferroni-corrected p-values were calculated using the QEA module of Metaboanalyst MSEA, based on the globaltest algorithm and a generalized linear model to estimate the Q-stat for each metabolite set.

Figure S5. LCM-RNAseq analysis of specific muscle fibers.

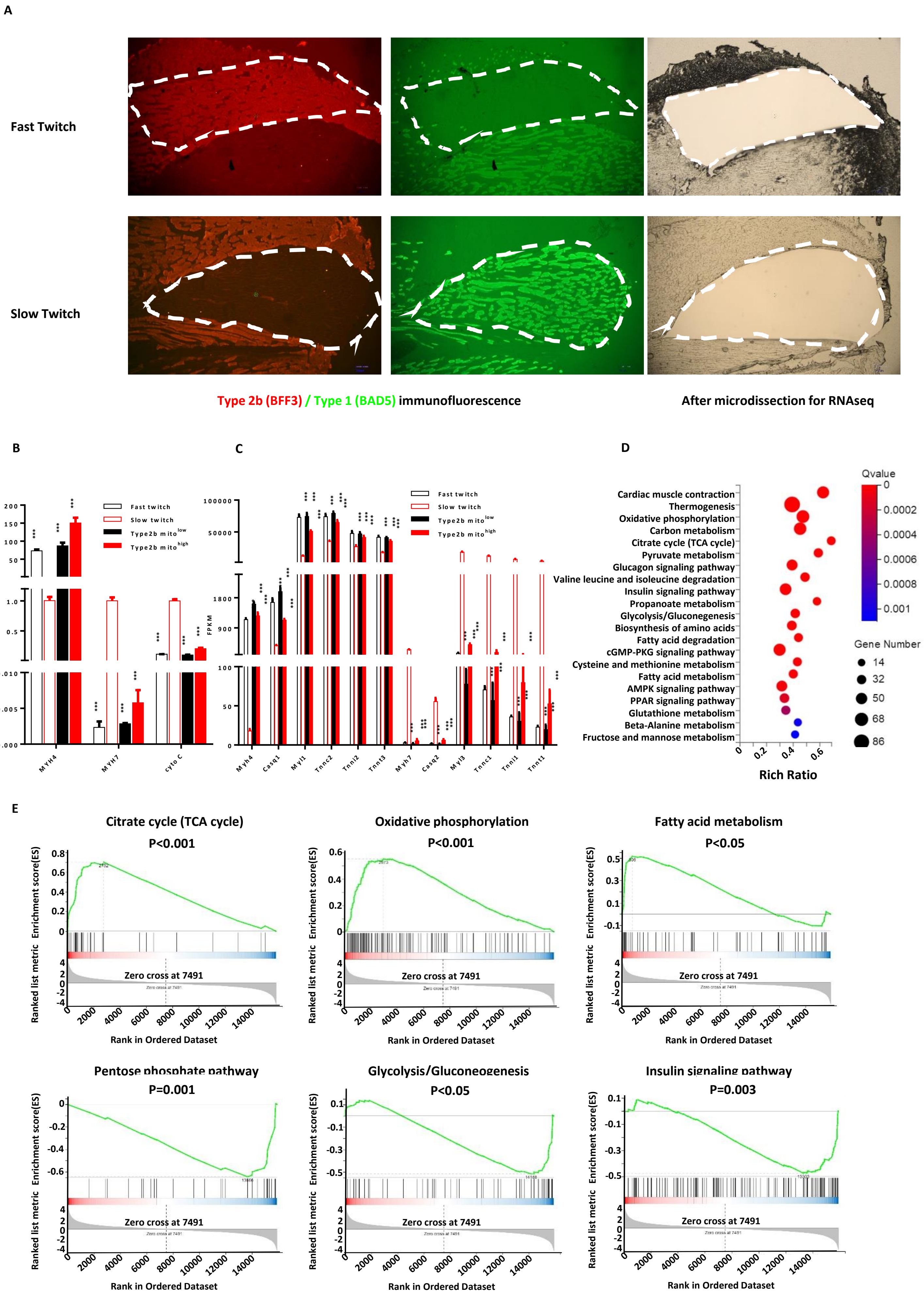

Figure S5. LCM-RNAseq analysis of specific muscle fibers.

A. Laser capture microdissection (LCM), in comparison with immunofluorescence images, accurately isolates gastrocnemius fast-twitch and soleus slow-twitch myofibers for RNAseq.

B. Quantitative real-time PCR (qRT-PCR) for Myh4, Myh7 and cytochrome C gene expression in fast-twitch, slow-twitch, type 2b mito^{high} and type 2b mito^{low} myofibers' RNA samples. (Mean±SEM, ***p<0.001, Student's t test).

C. Transcript abundance of fast-twitch myofiber markers (Myh4-Tnnt3) and slow-twitch myofiber markers (Myh7-Tnnt1). FPKM indicates Fragments Per Kilobase of transcript per Million fragments mapped in RNAseq (Mean±SEM, ***p<0.001, Student's t test).

D. Bubble chart of KEGG pathway enrichment analysis of differentially expressed genes (DEGs) in the slow-twitch myofibers, relative to fast-twitch myofibers. The Q-value is the corrected Fisher exact test p-value. Rich ratio refers to the ratio of the number of differential genes enriched in the pathway to the number of annotated genes. The greater the Rich ratio, the greater the degree of enrichment.

E. Gene Set Enrichment Analysis (GSEA) of the transcriptome of slow-twitch myofibers, relative to fast-twitch myofibers. GSEA signatures for the TCA cycle, Oxidative phosphorylation, and Fatty acid metabolism were significantly enriched in slow-twitch myofibers. GSEA signatures for the Pentose phosphate pathway, Glycolysis/Gluconeogenesis and Insulin signaling pathway were significantly enriched in fast-twitch myofibers. FWER-adjusted P-values for the GSEA test are calculated by permutation of statistics based on the Kolmogorov-Smirnov test.

Supplementary Tables

Table S1. Reproducibility of fingerprint metabolites by MSI

Table S2. Intersection of MALDI v.s. APMALDI

Table S3. Fingerprints of fast- and slow-twitch myofibers

Table S4. Intersection of MSI v.s. LC-MS
